# Classification of Southeast Asian mints (*Mentha* spp.) based on simple sequence repeat markers

**DOI:** 10.1270/jsbbs.21058

**Published:** 2022-03-09

**Authors:** Yuri Fukui, Moeko Saito, Natsuno Nakamura, Taichi Mizuno, Shuichi Sato, Mayu Tsukuda, Saori Nakaoka, Keita Tsuboi, Azusa Sasaki, Kouji Kuramochi, Panida Boonyaritthongchai, Nichapat Kaewmanee, Krit Thirapanmethee, Mullika Traidej Chomnawang, Bhanubong Bongcheewin, Thuy Linh Nguyen, Huong Lan Thi Nguyen, Huong Thi Le, Shigehisa Okamoto, Takako Nakamura, Yasushi Nakamura, Nakao Kubo

**Affiliations:** 1 Graduate School of Life and Environmental Sciences, Kyoto Prefectural University, 1-5 Hangi-cho, Shimogamo, Sakyo-ku, Kyoto 606-8522, Japan; 2 Faculty of Life and Environmental Sciences, Kyoto Prefectural University, 1-5 Hangi-cho, Shimogamo, Sakyo-ku, Kyoto 606-8522, Japan; 3 Department of Applied Biological Science, Faculty of Science and Technology, Tokyo University of Science, 2641 Yamazaki, Noda, Chiba 278-8510, Japan; 4 School of Bioresources and Technology, King Mongkut’s University of Technology Thonburi, Bangkhuntien, Bangkok 10150, Thailand; 5 Department of Microbiology, Faculty of Pharmacy, Mahidol University, 447 Sri-Ayuthaya, Rajathevi, Bangkok 10400, Thailand; 6 Department of Pharmaceutical Botany, Faculty of Pharmacy, Mahidol University, 447 Sri-Ayuthaya, Rajathevi, Bangkok 10400, Thailand; 7 Institute for Preventive Medicine and Public Health and Hanoi Medical University Hospital, Hanoi Medical University, 1 Ton That Tung, Dong Da, Hanoi 116001, Vietnam; 8 Department of Food Science and Biotechnology, Faculty of Agriculture, Kagoshima University, 1-21-24 Korimoto, Kagoshima 890-0065, Japan; 10 Planning Office, Kyoto Prefectural Agriculture, Forestry and Fisheries Technology Center, 9 Wakunari, Amarube-cho, Kameoka, Kyoto 621-0806, Japan; 11 Biotechnology Research Department, Kyoto Prefectural Agriculture, Forestry and Fisheries Technology Center, 74 Oji, Kitainayazuma, Seika-cho, Soraku-gun, Kyoto 619-0244, Japan

**Keywords:** classification, mint (*Mentha* spp.), simple sequence repeat (SSR), Southeast Asia

## Abstract

*Mentha* is a complex genus encompassing many species as a consequence of their interspecific hybridization and polyploidy. Southeast Asian mints have been poorly distinguished though they are widely used for culinary and medical purposes. In this study, we have analyzed Southeast Asian mints and known varieties as well as a related Lamiaceae species (*Nepeta* sp.) using simple sequence repeat (SSR) markers and leaf morphology. Two types of mints were clearly distinguished based on their venation pattern and leaf shape index. We developed 12 SSR markers that allowed good amplification in the *Mentha* and another Lamiaceae species. In the SSR-based phylogram, the *Mentha* lines could be delimited into groups I–VI. The Southeast Asian mints divided into groups I and II, and the phylogram separated most of the available species, with groups I and II containing the known species *M.* × *cordifolia* and *M. arvensis*, respectively. The separation of the two groups was supported by a population structure analysis. The SSR markers developed in this study enabled the simultaneous classification of mints and will help improve our understanding of the genetic composition of known mint varieties and as yet unclassified Southeast Asian mints.

## Introduction

Mints (*Mentha* spp.) belong to the Lamiaceae family. *Mentha* species are propagated vegetatively and through seed. Many hybrids have been generated from natural pollination and cross breeding. In addition to the basic diploids, polyploids have also been reported (see Kimura 2020, Tucker and Naczi 2007, Supplemental Text 1). The genus *Mentha* includes species complex as a consequence of natural interspecific hybridization and polyploidy. In Southeast Asia, mints are widely used for culinary and medical purposes. We previously have focused on an anticarcinogenic compound in a Southeast Asian mint (Nakamura *et al.* 2014) and conducted field collections. However, there is a paucity of information on their origins. Mints have been poorly distinguished in the markets and are usually identified by local names (Cantoria 1968, Lợi 1986, Ngearndee 1987).

The classification of *Mentha* species has traditionally been conducted using their morphological and cytological features as well as their chemical compositions (see Tucker and Naczi 2007 for a review, Supplemental Text 1). However, these analyses are often tedious as there are continuous variations and/or environmental effects. Mints have been classified using several molecular markers such as internal transcribed spacer (ITS) region, restriction fragment length polymorphism, randomly amplified polymorphic DNA (RAPD), amplified fragment length polymorphism (AFLP), and inter-simple sequence repeat (ISSR) (Yaghini *et al.* 2020, Supplemental Text 1). Simple sequence repeats (SSRs) or microsatellites are DNA repeats consisting of 1–6 nucleotide motifs (see Merritt *et al.* 2015 for a review, Supplemental Text 1). SSRs are frequently used as DNA markers because of their abundance in eukaryotic genomes, ease of detecting their polymorphisms, and relatively high levels and reproductivity of the polymorphism). SSR markers have been developed and used for the classification of mints (Kumar *et al.* 2015, Vining *et al.* 2019). However, because of extremely high levels of sequence-specificity, SSRs tend to have low transferability among different species. Intron-targeted polymorphisms, also called intron length polymorphism (ILP) markers, have also been utilized as molecular markers. ILP markers detect indels within intragenic regions using primer pairs that anneal to conserved exon regions to amplify its flanking intron (Choi *et al.* 2004). They are sequence-specific and have relatively high levels of polymorphism as well as good transferability among related species. As SSR and ILP markers share similar characteristics as valuable molecular markers, their merits can be integrally utilized to develop improved markers to study mint genetic diversity.

In this study, we have analyzed Southeast Asian mints and known varieties using SSR markers and leaf morphology. To successfully classify multiple *Mentha* species, we searched for SSRs introns of the horse mint (*M. longifolia* (L.) Huds.) genome (Vining *et al.* 2017) and designed primer pairs that anneal to the conserved exon regions and amplify SSR-containing introns. The objective of this study was to classify the Southeast Asian mints using SSR markers that enable simultaneous classification of different mint species. The proposed relationships of the Southeast Asian mints and the redefined classifications of some of the mint varieties are discussed.

## Materials and Methods

### Plant materials

A total of 120 (119 *Mentha* and one *Nepeta*) lines were investigated in this study (Supplemental Table 1). For the *Mentha* lines, 86 were collected from Laos, Myanmar, Thailand, and Vietnam (Supplemental Fig. 1), two were collected from Japan, 22 were commercially available varieties in Japan, and seven were germplasms obtained from the genebanks. The material transfer for the Southeast Asian mints to Japan was done prior to the enforcement of international rules such as the Convention on Biological Diversity and the Nagoya Protocol on Access and Benefit Sharing. Two F_1_ offsprings were obtained from a cross between Spearmint [Pot] and Apple mint [Pot] to confirm the inheritance of SSR alleles. Each line (at least five individuals per line) was vegetatively propagated and maintained in pots in a glasshouse at Kyoto Prefectural Agriculture, Forestry and Fisheries Technology Center (Soraku-gun, Kyoto) under natural sunlight and a minimum temperature of 2°C.

### Morphological and cytological analyses

Because of the similarity in appearance of mint lines in the same group, leaf venation patterns for 83 selected lines (77 Southeast Asian mints, plus three spearmints and three apple mints; Supplemental Table 2) were visually inspected and classified as either a parallel or reticulate venation arrangement (Supplemental Fig. 2). The leaf shape index (LSI: length/width) was determined using five representative, fully expanded leaves chosen from each line. The Tukey-Kramer multiple comparison test was performed using BellCurve for Excel 3.21 (Social Survey Research Information, Tokyo). For the cytological analysis, root tips were harvested from nine representative lines (#056, #114, #128, #135, Apple mint [Pot], Horse mint-PI557757, Kentucky colonel [Kur], Ryokubi-JP176285, and Spearmint [Pot]) and treated as reported previously (Tjio and Levan 1950). Their chromosomes were observed in bright field by a BX51 microscope (Olympus Corporation, Tokyo).

### DNA extraction and development of SSR markers

Genomic DNA was isolated as previously reported (Kubo *et al.* 2019) from a single representative individual for each line. The genomic DNA of Horse mint-PI557757 used for the genome sequencing (Vining *et al.* 2017) was kindly supplied by Dr. K. Vining (Oregon State University, Corvallis, Oregon). Two specimens of *M.* × *cordifolia* Opiz ex Fresen. (syn. *M.* × *villosa* Huds., a hybrid of spearmint and apple mint) from Indonesia were provided by the Naturalis Biodiversity Center (Leiden). We searched for intron polymorphic SSRs with ≥6 dinucleotide repeats within annotated gene regions of the horse mint genome. Each primer pair was basically designed within predicted exon regions that sandwiched an intron and amplified a ≤400 bp amplicon (Supplemental Fig. 3). The two previously-reported SSR markers EMM_007 and EMM_049 (Kumar *et al.* 2015) were modified in accordance with the genomic sequences.

### Detection of alleles, estimation of genetic parameters, construction of a phylogram and population structure analysis

SSR fragments were amplified by polymerase chain reaction with fluorescently-labeled primers as reported previously (Kubo *et al.* 2019). The SSR alleles were recorded as binary data (presence/absence of band = 1/0) because more than two alleles were detected for each line in most cases. Genetic parameters were estimated with Popgene 1.32 (Yeh *et al.* 1997) for groups with more than two individuals. Construction of a neighbor-joining (NJ) phylogram and bootstrap analysis were performed using TREECON 1.3b (Van de Peer and De Watcher 1994). The *Nepeta* line was used as an outgroup according to Li *et al.* (2016). Detection of a hierarchical genetic population structure was investigated using Structure 2.3.4 (Hubisz *et al.* 2009) as reported previously (Kubo *et al.* 2019).

## Results

### Morphological observations

**Fig. 1.**
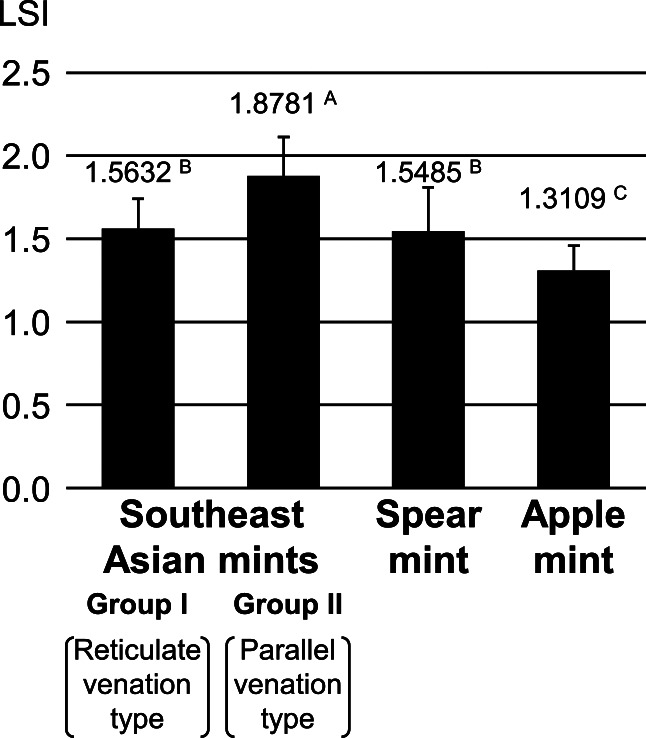
Average leaf shape index (LSI) values for four mint groups (group I, group II, spearmint, and apple mint). See Fig. 2 for the nomenclature of each group and Supplemental Table 2 for more detailed information. Average LSI values for each group are shown on the filled bar. Standard deviation is indicated with an error bar. Different uppercase letters indicate significant deviations (*P* < 0.01) from the other groups as determined by the Tukey-Kramer multiple comparison test.

Leaf venation pattern was observed in 83 mint lines (Supplemental Table 1, Supplemental Fig. 1). There were two clear types of venation patterns in the 77 Southeast Asian lines, as 58 and 19 had reticulate and parallel venations, respectively (Supplemental Table 2, Supplemental Fig. 2A, 2B). Spearmints and apple mints had reticulate venation. The average LSI for the above 19 lines was significantly larger than that of the other groups investigated (Fig. 1, Supplemental Table 2). The average value of the spearmints was similar to that of the 58 lines, and the LSI of the apple mints was the smallest.

### Development of SSR markers and the genetic diversity of the mint lines

**Fig. 2.**
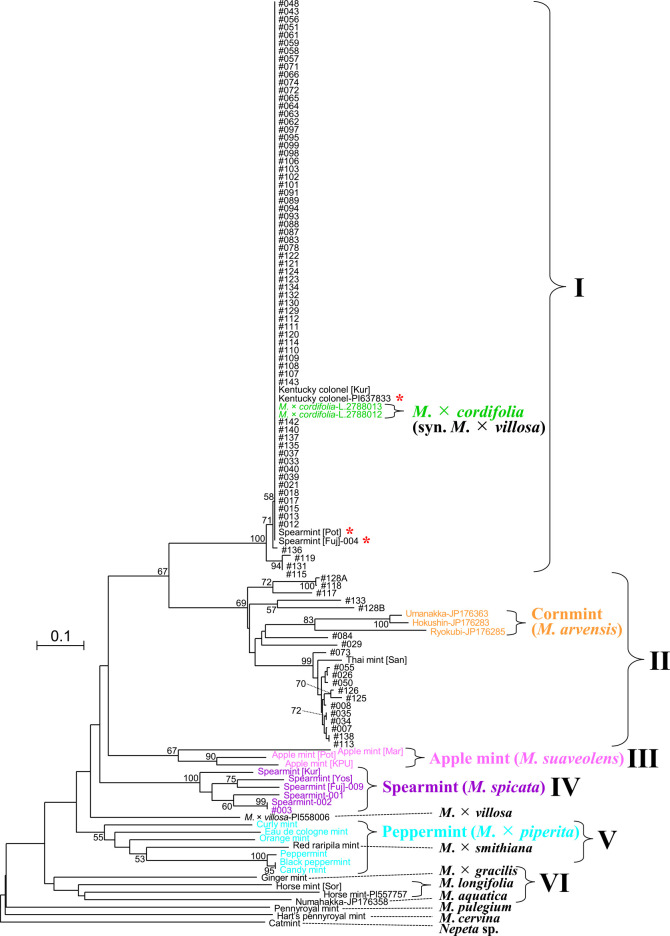
A neighbor-joining (NJ) phylogram of 119 mint lines (*Mentha* spp.) and another Lamiaceae species (*Nepeta* sp.) constructed based on 12 SSR markers. Species names are shown for representative lines. Six potential groups are indicated with roman numerals (I–VI). The numbers in each node are the bootstrap values (≥50%). The scale bar indicates the genetic distance (Nei and Li 1979). The red asterisks indicate species classifications identified in this study as suspicious. See online article for color version of this figure.

We initially tested the existing *Mentha* SSR markers investigated by Kumar *et al.* (2015), but they yielded unstable amplification depending on the species (data not shown), probably because of low cross-species amplification. We consequently developed SSR markers (Supplemental Fig. 3) by designing primers to anneal to the more conserved exons rather than the intergenic regions that are generally targeted for SSRs. We developed 12 SSR markers, 10 newly developed (mostly ILP-SSRs) and two modified SSRs (Table 1). These markers provided good amplification of the *Mentha* and *Nepeta* species tested. They often produced more than two bands (Table 1, Supplemental Table 3), which was unsurprising as many *Mentha* species have a hybrid origin. Indeed, the mints studied are likely to include tetraploids and octoploids in addition to diploids (Supplemental Fig. 4, Supplemental Table 3). Therefore, the alleles were recorded as binary data (1 and 0). Inheritance of detected SSR alleles was confirmed in the F_1_ offsprings as the offsprings always shared alleles with their parents (Supplemental Table 4, red, blue, and gray boxes). The genetic diversity estimates for accession in group I, which was identified in the phylogram (Fig. 2), were the lowest of the five groups examined (Supplemental Table 5).

### Phylogram construction and population structure detection using SSR markers

An NJ phylogram created using the 12 SSR markers separated the lines into groups I–VI (Fig. 2). The Southeast Asian mints divided into groups I and II. The lines with reticulate and parallel venation types (Supplemental Table 2) belonged to groups I and II, respectively. Group I contained 67 Southeast Asian mints, two commercial spearmints (Spearmint [Fuj]-004 and Spearmint [Pot]), two *M.* × *cordifolia*, and two Kentucky colonel. Of these, 69 lines excluding #115, 119, 131, and 136 had identical genotypes. Group II contained 19 Southeast Asian mints, one commercial mint (Thai mint [San]), and three cornmints (Hokushin-JP176283, Ryokubi-JP176285, and Umahakka-JP176363). The Southeast Asian mints in group II were mostly derived from the northeastern part of the Indochinese Peninsula (Supplemental Fig. 1, orange color), and their distribution showed no apparent pattern. Groups III, IV, and V included apple mints, the remaining spearmints, and peppermints (plus a red raripila mint), respectively. Group VI was composed of ginger, water, and horse mints with <50% bootstrap support.

**Fig. 3.**
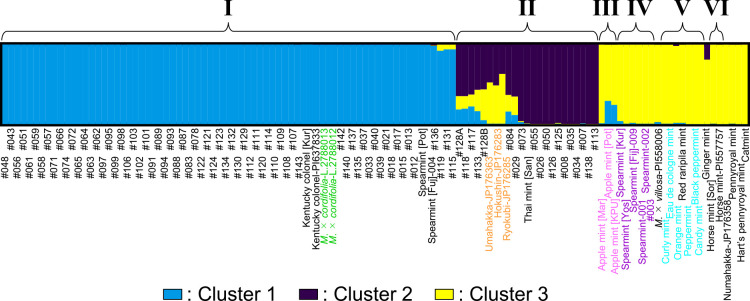
Genetic structure of the 120 lines based on 12 SSR markers. The result at the most suitable subpopulation number (*K* = 3) is shown. Sample names are beneath the barplots. The colors of the line names are in accordance with those in Fig. 2. The colors of the barplots correspond to the three assumed clusters (1–3). The six potential groups (I–VI) observed in Fig. 2 are indicated at the top. See online article for color version of this figure.

The separation of the Southeast Asian mints into groups I and II was confirmed by a hierarchical population structure. At the most suitable number of subpopulations (*K*) = 3 (Supplemental Table 6, underline), group I lines mostly occupied cluster 1, whereas group II lines constituted over half of cluster 2 (Fig. 3, blue and dark purple colors, respectively). The observed separation was supported in the other *K* (2 and ≥4, Supplemental Fig. 5). Groups III–VI shared cluster 2 with group II at *K* = 2, whereas at *K* ≥ 3 they were mostly represented by clusters different from those occupied by groups I and II.

## Discussion

In this study, we classified Southeast Asian mints as well as commercial varieties. First, leaf morphology was examined in selected lines, and two main types were revealed (Fig. 1, Supplemental Table 2). We then used intronic SSR markers with high transferability across multiple species. Each SSR marker detected more than two bands in a given sample. We assume the possibility of amplifying two or more duplicate loci (Holland *et al.* 2001) was low because the allele number of SSR markers corresponded well to predicted ploidy levels (Supplemental Table 3, red letters) and no incongruence in the inheritance of alleles was detected (Supplemental Table 4). Although the genotyping was similar to that used for other types of dominant markers (AFLP, ISSR, RAPD, etc.), the SSR markers used in this study had an advantage as their loci were known in advance. Most of the species belonging to groups I–V were divided into different groups with moderate to high bootstrap values (55%–100%) in the phylogram (Fig. 2), demonstrating a successful classification of multiple species, although groups III–VI were not well-resolved in the population structure (Fig. 3, Supplemental Fig. 5) for unknown reasons. A red raripila mint was included in the peppermint group, probably because they share two parents (*M. aquatica* and *M. spicata*).

Using the leaf morphology and SSR markers, Southeast Asian mints were divided into groups I and II, including the known species *M.* × *cordifolia* and *M. arvensis*, respectively. Group I mints had identical or closely-related genotypes to *M.* × *cordifolia* and Kentucky colonel. A previous report found that *M.* × *cordifolia* was cultivated in Java, Philippines, and Thailand (Mỡi 1999). Its morphological characteristics and ITS sequence have been characterized (Cantoria 1968, Ngearndee 1987, Sitthithaworn *et al.* 2009). However, to the best of our knowledge, no comprehensive comparison of the Southeast Asian mints has yet been conducted. Although *M.* × *cordifolia* is currently classified as a synonym of *M.* × *villosa*, their relationships are confused (Kimura 2020). The placement of spearmints (Spearmint [Fuj]-004 and Spearmint [Pot]) in group I (Fig. 2, red asterisks) would constitute another case of such confusion. Species identification of Kentucky colonel is also confused (see Kimura 2020, Tucker and Naczi 2007). It has been classified as a spearmint (*M. spicata*; Tucker and Naczi 2007) or *M.* × *villosa* (Mỡi 1999). In this study, two independent Kentucky colonel lines had identical genotypes to *M.* × *cordifolia*, one of which is being classified to *M. spicata* (Fig. 2, red asterisk). The *M.* × *cordifolia* and Kentucky colonel were separated from the spearmint group and *M.* × *villosa*-PI558006 (Fig. 2). While the classification of *M.* × *villosa* is still controversial, the present data could help clarify the relationship between *M.* × *cordifolia* and Kentucky colonel. The group I mints were closely related to each other as seen from their short branch lengths in the phylogram (Fig. 2) and low genetic diversity (Supplemental Table 5). The group I mints sometimes flowered during our cultivation in the glasshouse but no mature seed was obtained (data not shown). A few seeds could have been obtained by hand cross-pollination after many trials (Supplemental Table 4). There was no contradiction to the report that *M.* × *cordifolia* never flowered, and thus is propagated vegetatively in Southeast Asia (Mỡi 1999). Group II contained cornmints (*M. arvensis*). The Japanese cornmint cultivar Ryokubi has been introduced to Thailand and is called “So Wo 1” (Chomochalow *et al.* 1976). Ryokubi and the 19 lines of Southeast Asian mint belonged to group II, confirming their genetic relationship. Genetic differences between Ryokubi and the Southeast Asian lines (see branch lengths in Fig. 2) may suggest the occurrence of seed propagation in the Southeast Asian lines and/or multiple introductions. Although cornmints can be classified into two species (*M. arvensis* and *M. canadensis* L.), both species are difficult to identify, and their identification often leads to disagreements (Semenova *et al.* 2019). We have here treated the cornmint lines as a single species, *M. arvensis*.

Mint was reportedly brought to Thailand during the reign of King Rama III (1824–1851) by an Italian (Hongwiwat 2002), and much later, Ryokubi was introduced there (Chomochalow *et al.* 1976). This history of two introductions might be reflected in the two genetically- and morphologically-distinct groups (I and II) that were identified in this study (Figs. 1–3).

In conclusion, we have developed SSR markers that enable the simultaneous classification of various mint species. The information presented here will help us understand the genetic composition of commercial varieties and as yet-unclassified Southeast Asian mints. These data will contribute to the understanding of the genetic diversity and efforts towards the improvement of mint and other Lamiaceae species in Southeast Asia and other countries.

## Author Contribution Statement

TN, YN and NK designed the study. SS, PB, NK, KT, MTC, BB, TLN, HLTN, HTL, SO, TN and YN collected samples. YF, MS, NN, TM, SS, MT, SN, KT, AS, KK and NK maintained the plant samples and performed the experiments. YF and NK drafted the manuscript. YN revised the manuscript. All authors read and approved the final manuscript.

## Supplementary Material

Supplemental Figures

Supplemental Tables

Supplemental Text

## Figures and Tables

**Table 1.** List of simple sequence repeat (SSR) markers developed and modified in this study

Marker name	Primer sequence (5ʹ-to-3ʹ)	Repeat motif*^a^*	Expected size (bp)*^b^*	Allelic range observed (bp)	Allele number per line observed	Sequence origin and nucleotide positions*^c^*
*SSR markers developed from the mint genome data*						
Cont017-gene0.11	Forward: ACTATCATCACCGGCATTATCAC	(CT)_11_(CA)_8_	149	134–201	1–7	Contig17: 20,453-20,305
	Reverse: TTTGATGTCGAAATAATCACTCCC					
Cont028-gene0.2	Forward: AATAGGGAGTCTGCTGCTAGGT	(TC)_8_	199	116–235	1–8	Contig28: 14,579-14,381
	Reverse: CAGTGACTCCAACTCAACGGTATA					
Cont030-gene0.5	Forward: CAGCATATTGTTGAAGTGAAGCCACA	(TC)_12_	133	127–195	1–7	Contig30: 19,523-19,391
	Reverse: ACTTTCTCCGCAGCCAAACCTTT					
Cont039-gene0.9	Forward: ACATCTCCCGAATTATCTGTCCAT	(CT)_11_	151	125–257	1–8	Contig39: 8,306-8,156
	Reverse: GCTGTATAATACTTTGTGTTGATTGTCC					
Cont040-gene0.3	Forward: CCAGAGTCCAACATAAGAATTTAGTTA	(TG)_9_	254	160–384	1–6	Contig40: 7,922-7,669
	Reverse: AGGTTCCTTGCAAGCTCCAATGAA					
Cont055-gene0.4	Forward: AAGAATTGAGAGCTGTGCTTCCAT	(TG)_6_gg(GT)_4_	181	152–375	1–7	Contig55: 13,384-13,564
	Reverse: CTCAAAGTTCTGAATTTCAATGTGG					
Cont119-gene0.0	Forward: ACCGTAGTACTATTATTGTTCCTGC	(TG)_6_	216	218–488	1–2	Contig119: 3,202-3,417
	Reverse: GGGCTTCAGAGTTCTGCTGAT					
Cont130-gene0.5	Forward: CATTCGACTTGAAGGGCTGG	(TA)_5_t(TA)_10_ (GATA)_5_	327	180–400	1–4	Contig130: 8,249-8,575
	Reverse: CAGGGAACAATTCCGGCTCATA					
Cont138-gene0.5	Forward: TCTCACAAACAAATCGGCACTTCT	(TG)_10_	196	162–304	1–6	Contig138: 12,869-12,674
	Reverse: TGGGTAGTCTAGGAACAAGTTTTC					
Cont171-gene0.6	Forward: GCCTGTGCTTCTTCATTACTTATC	(CT)_9_(CA)_9_	143	134–196	1–6	Contig171: 7,157-7,015
	Reverse: TTGTTGCCACTCTTAGTTCTAGG					
*SSR markers modified from Kumar et al. (2015) based on the mint genome data*						
EMM_007-Mp	Forward: CTTTTGCAGTTATCAATTGTTTCACCA	(CT)_6_tcatt(TTC)_5_ a(TC)_8_(AC)_13_	138	108–578	1–7	Contig23404: 17,981-17,844
	Reverse: ATTCCATAGCATGATGCAGCCAT					
EMM_049-Mp	Forward: GAAGCAGGCCCAACCACC	(CT)_6_	98	98–434	1–6	Contig54312: 3,263-3,360
	Reverse: GAAATTAACAACAGTTCTGAATACTCGT					

*^a^* Repeat numbers are derived from the mint genome (Vining *et al.* 2017) or cDNA sequence (Kumar *et al.* 2015). *^b^* Size expected from the mint genome sequence (Vining *et al.* 2017) . *^c^* Name of contig and nucleotide positions (bp) in the mint genome corresponding to of the amplicon of each SSR marker.

## References

[B1] Cantoria, M. (1968) Physiological and chemical studies for the identification of Philippine mint. Philipp J Sci 97: 277–283.

[B2] Choi, H.K., D. Kim, T. Uhm, E. Limpens, H. Lim, J.H. Mun, P. Kalo, R.V. Penmetsa, A. Seres, O. Kulikova et al. (2004) A sequence-based genetic map of *Medicago truncatula* and comparison of marker colinearity with *M. sativa*. Genetics 166: 1463–1502. 15082563 10.1534/genetics.166.3.1463 PMC1470769

[B3] Chomochalow, N., P. Buransilpin, N. Pichitakul, A. Euraree and A. Pangspa (1976) Hill-tribe mint production and processing. Thai J Agric Sci 9: 127–144.

[B4] Holland, J.B., S.J. Helland, N. Sharopova and D.C. Rhyne (2001) Polymorphism of PCR-based markers targeting exons, introns, promoter regions, and SSRs in maize and introns and repeat sequences in oat. Genome 44: 1065–1076. 11768210

[B5] Hongwiwat, T. (2002) Saranae (mint). *In*: Vegetable Encyclopedia. Sangdad Publishing, Bangkok, pp. 156–157 (in Thai).

[B6] Hubisz, M.J., D. Falush, M. Stephens and J.K. Pritchard (2009) Inferring weak population structure with the assistance of sample group information. Mol Ecol Resour 9: 1322–1332. 21564903 10.1111/j.1755-0998.2009.02591.x PMC3518025

[B7] Kimura, M. (2020) Botany and cultivation of mint (Mint no Shokubutsugaku to Saibai). Medical Herb: Japan Medical Herb Association News 52: 16–23 (in Japanese).

[B8] Kubo, N., H. Ueoka and S. Satoh (2019) Genetic relationships of heirloom turnip (*Brassica rapa*) cultivars in Shiga Prefecture and other regions of Japan. Hort J 88: 471–480.

[B9] Kumar, B., U. Kumar and H.K. Yadav (2015) Identification of EST-SSRs and molecular diversity analysis in *Mentha piperita*. Crop J 3: 335–342.

[B10] Li, B., P.D. Cantino, R.G. Olmstead, G.L. Bramley, C.L. Xiang, Z.H. Ma, Y.H. Tan and D.X. Zhang (2016) A large-scale chloroplast phylogeny of the Lamiaceae sheds new light on its subfamilial classification. Sci Rep 6: 34343. 27748362 10.1038/srep34343 PMC5066227

[B11] Lợi, T.Đ. (1986) Bạc hà (mint). *In*: Medicinal Plants and Drugs of Vietnam, 5th edn. Science and Technics Publishing House, Hanoi, pp. 601–605 (in Vietnamese).

[B12] Merritt, B.J., T.M. Culley, A. Avanesyan, R. Stokes and J. Brzyski (2015) An empirical review: characteristics of plant microsatellite markers that confer higher levels of genetic variation. Appl Plant Sci 3: 1500025. 10.3732/apps.1500025 PMC4542939 26312192

[B13] Mỡi, Đ.L. (1999) *Mentha arvensis* L. *In*: de Padua, L.S., N. Bunyapraphatsara and R.H.M.J. Lemmens (eds.) Plant Resources of South-east Asia. No. 12(1), Medicinal and Poisonous Plants 1. Backhuys Publishers, Leiden, pp. 344–349.

[B14] Nakamura, Y., Y. Hasegawa, K. Shirota, N. Suetome, T. Nakamura, M.T. Chomnawang, K. Thirapanmethee, P. Khuntayaporn, P. Boonyaritthongchai, C. Wongs-Aree et al. (2014) Differentiation-inducing effect of piperitenone oxide, a fragrant ingredient of spearmint (*Mentha spicata*), but not carvone and menthol, against human colon cancer cells. J Funct Foods 8: 62–67.

[B15] Nei, M. and W.H. Li (1979) Mathematical model for studying genetic variation in terms of restriction endonucleases. Proc Natl Acad Sci USA 76: 5269–5273. 291943 10.1073/pnas.76.10.5269 PMC413122

[B16] Ngearndee, P. (1987) The pharmacognostical study on the leaf of “sa-ra-nae” (*Mentha cordifolia* Opiz. family labitae). Bulletin of the Department of Medical Sciences 29: 39–48 (in Thai with English summary).

[B17] Semenova, M.V., O.L. Enina and O.V. Shelepova (2019) Intra- and interspecific variability of *Mentha arvensis* L. and *M. canadensis* L. Vavilovskii Zhurnal Genet Selektsii 23: 1067–1075.

[B18] Sitthithaworn, W., S. Vimolmangkang, C. Chittasupho, D. Petcheunsakul and S. Apa-adul (2009) Pharmacognostic investigation of the leaves of *Mentha cordifolia* and its DNA fingerprints. Thai Pharmaceutical and Health Science Journal 4: 9–14.

[B19] Tjio, J.H. and A. Levan (1950) The use of oxyquinoline in chromosome analysis. Anales de la Estación Experimental de Aula Dei 2: 21–64.

[B20] Tucker, A.O. and R.F.C. Naczi (2007) *Mentha*: an overview of its classification and relationships. *In*: Lawrence, B.M. (ed.) Mint: The Genus *Mentha*. CRC Press, Boca Raton, Florida, pp. 1–39.

[B21] Van de Peer, Y. and R. De Wachter (1994) TREECON for Windows: A software package for the construction and drawing of evolutionary trees for the Microsoft Windows environment. Bioformatics 10: 569–570. 10.1093/bioinformatics/10.5.569 7828077

[B22] Vining, K.J., S.R. Johnson, A. Ahkami, I. Lange, A.N. Parrish, S.C. Trapp, R.B. Croteau, S.C.K. Straub, I. Pandelova and B.M. Lange (2017) Draft genome sequence of *Mentha longifolia* and development of resources for mint cultivar improvement. Mol Plant 10: 323–339. 27867107 10.1016/j.molp.2016.10.018

[B23] Vining, K.J., I. Pandelova, K. Hummer, N. Bassil, R. Contreras, K. Neill, H. Chen, A.N. Parrish and B.M. Lange (2019) Genetic diversity survey of *Mentha aquatica* L. and *Mentha suaveolens* Ehrh., mint crop ancestors. Genet Resour Crop Evol 66: 825–845.

[B24] Yaghini, H., M.R. Sabzalian, M. Rahimmalek, T. Garavand, A. Maleki and A. Mirlohi (2020) Seed set in inter specific crosses of male sterile *Mentha spicata* with *Mentha longifolia*. Euphytica 216: 46.

[B25] Yeh, F.C., R.C. Yang, T.B.J. Boyle, Z.H. Ye and J.X. Mao (1997) POPGENE, the user-friendly shareware for population genetic analysis. Molecular Biology and Biotechnology Centre, University of Alberta, Edmonton.

