# Supplementary material for: Classification of Southeast Asian mints (Mentha spp.) based on simple sequence repeat markers
Source: Breed Sci. 2022 Mar 9;72(2):181–7. doi: 10.1270/jsbbs.21058 (PMC9522532; doi:10.1270/jsbbs.21058)
Supplement: Supplementary file 1 — Supplemental Figures [file 72_181_s1.pdf]

doi: 10.1270/jsbbs.21058

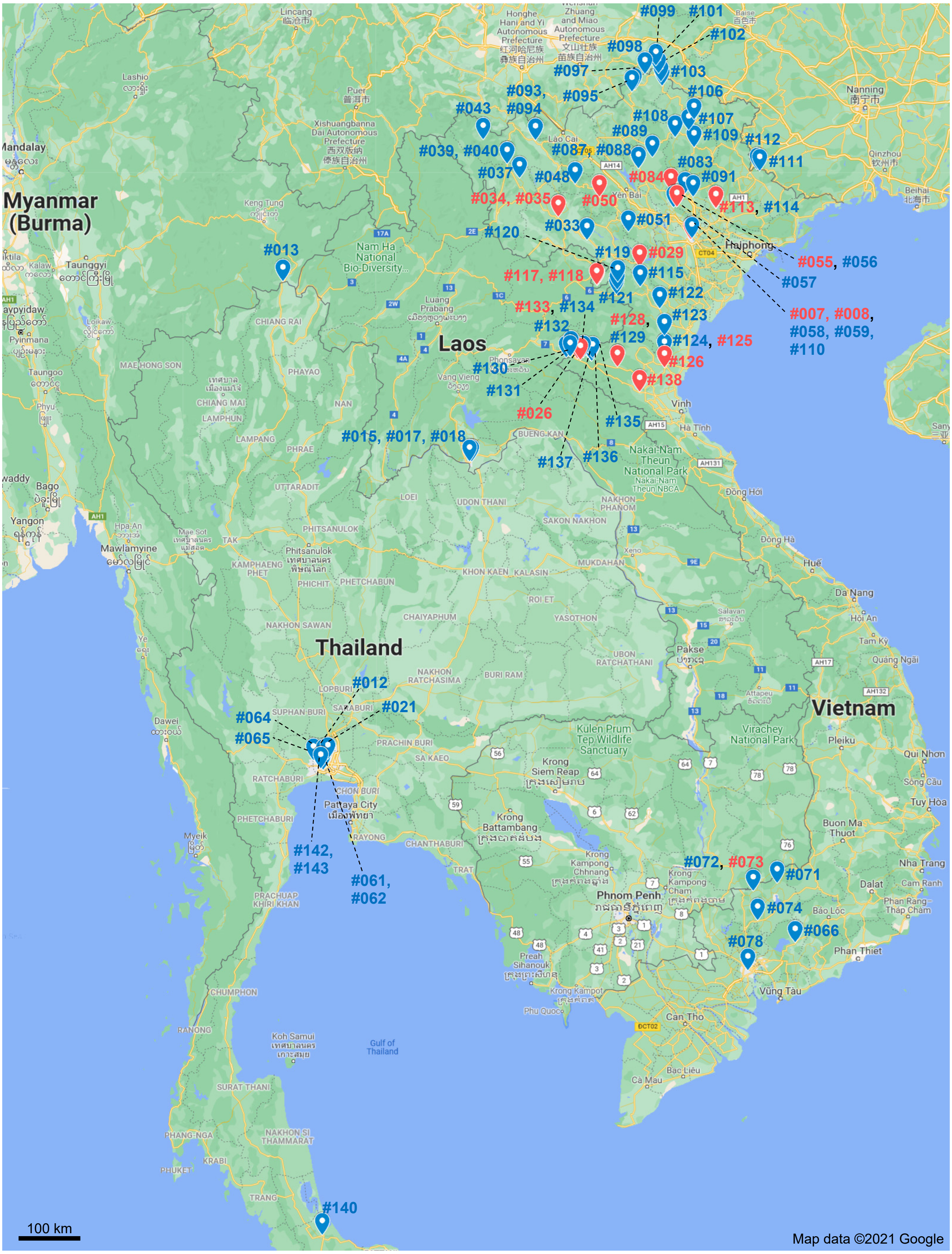


**Supplemental Fig. 1.** Collection sites of mint lines in Southeast Asian countries. Geographic positions collected and line names are indicated with pin marks on the map (see Supplemental Table 1 for latitude and longitude of each collection site). Lines belonging to groups I and II (Fig. 2) are indicated with blue and orange colors, respectively

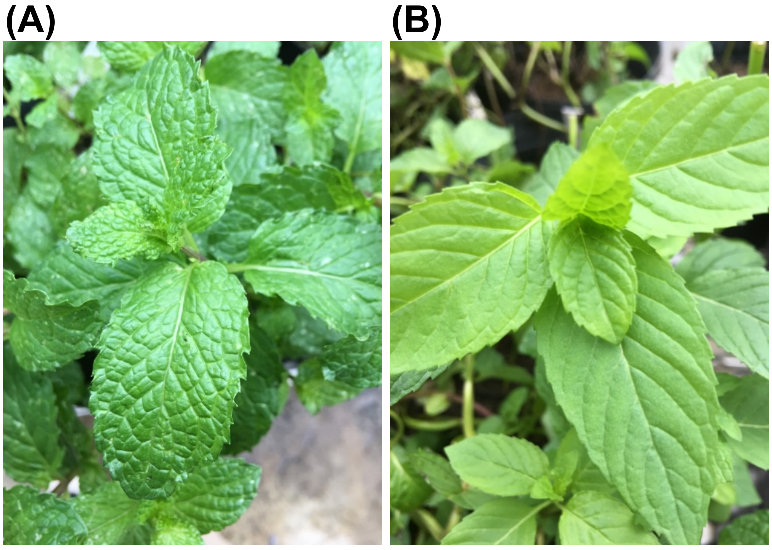


**Supplemental Fig. 2.** Leaf venation patterns of mint. (A) Reticulate (line #57) or (B) parallel venation pattern (line #84) is shown as a representative.

Contig17
Gene ID: augustus_masked-Contig17-abinit-gene-0.11 (homology to methylthioribose-1-phosphate isomerase)
Upper case: putative exons (homology to *M.* × *piperita* cDNA (AW255572))

```
     21170     21160     21150     21140     21130     21120     21110     21100     21090     21080     21070     21060
tttgttttattgattgaacagtctagcaacagctggataTGGAACTGCTCTAGGCGTGATCCGTGCCCTTCATGAGAATGGAGTCCTAGAAAGGGCTTACTGCACCGAAACACGGCCTTT
                                         G  T  A  L  G  V  I  R  A  L  H  E  N  G  V  L  E  R  A  Y  C  T  E  T  R  P  F

     21050     21040     21030     21020     21010     21000     20990     20980     20970     20960     20950     20940
CAATCAAgttagttttattcgtttgacatgttgaagaaaatatggttgcatagtatggtctctagaaaggatagaactagtagaattgactgtagttcgtgttatccattcattaatcgc
 N  Q

    20930     20920     20910     20900     20890     20880     20870     20860     20850     20840     20830     20820
ctcagGGCTCAAGACTGACAGCTTTTGAGTTGGTACATGACAATATTCCTGCAACTCTAGTAGCTGATTCTGCAGCAGCAGCATTGATGAGAGCTGGGAAGGTGAACGCTGTGGTTGTCG
     G  S  R  L  T  A  F  E  L  V  H  D  N  I  P  A  T  L  V  A  D  S  A  A  A  A  L  M  R  A  G  K  V  N  A  V  V  V  G

     20810     20800     20790     20780     20770     20760     20750     20740     20730     20720     20710     20700
GGGCTGATCGTGTGGCAGCCAATGgtatgtatctgaaaacttctgatgtaactcttattcttagaaaatatccaaataagtttatgagtgggtgtgtaagtagtaattgagttcctcgat
  A  D  R  V  A  A  N  G

     20690     20680     20670     20660     20650     20640     20630     20620     20610     20600     20590     20580
ttactagGTGATACTGCTAATAAGATCGGAACTTACAGCCTTGCCCTGTCAGCAAAGCATCACGGTATTCCTTTTTATGTGGCTGCTCCTCTTACTTCTGTCGATTTGTCCATTTCCTCT
         D  T  A  N  K  I  G  T  Y  S  L  A  L  S  A  K  H  H  G  I  P  F  Y  V  A  A  P  L  T  S  V  D  L  S  I  S  S

     20570     20560     20550     20540     20530     20520     20510     20500     20490     20480     20470     20460
GGCCAAGAAATCGTTATAGAAGAAAGGTCACCTAAAGAACTACTCCATGCTCGCGGAGGACTGGGAGAGCAAGTGGCTGCTTCCGGGATTTCTGTCTGGAATCCTGCCTTCGATGTCACA
G  Q  E  I  V  I  E  E  R  S  P  K  E  L  L  H  A  R  G  G  L  G  E  Q  V  A  A  S  G  I  S  V  W  N  P  A  F  D  V  T

      Cont017-gene0.11 F  -->
     20450     20440     20430     20420     20410     20400     20390     20380     20370     20360     20350     20340
CCAGCTACTATCATCACCGGCATTATCACAGAAAAGgtactctctctctctctctctctctcacacacacacacacagtcacacacgctgattctctccttgttcctgtacagGGAGTGG
P  A  T  I  I  T  G  I  I  T  E  K                                                                             G  V  V

          <--  Cont17-gene0.11 R
     20330     20320     20310     20300     20290     20280     20270     20260     20250     20240     20230     20220
TGACGAAAGATGGGAGTGATTATTTCGACATCAAAAGccggggtcgctcaaattgcttgatcaggctacttttatttctaagaaaaggttttggcttttttttttttttttttttttgc
  T  K  D  G  S  D  Y  F  D  I  K  S

     20210     20200     20190     20180     20170     20160     20150     20140     20130     20120     20110     20100
tcaaggaatggttgatagttatacaatgtattttgtaggctattatcttcaatttcttgttatattttcaatttagaaaggtgctcctttaaagtaagctgtttgcgcctttctgcagag
```

**Supplemental Fig. 3.** Sequences used for the design of 12 SSR markers in this study. Contig name of the *Mentha longifolia* genome (Vining *et al*. 2017), in which the amplicon of each SSR marker is included, is shown at the top. The gene ID annotated in the Mint Genomic Resource website (http://langelabtools.wsu.edu/mgr/) is also shown for each contig. Nucleotide positions correspond to those in each contig. Nucleotide sequences corresponding the exons are indicated with uppercase letters and their predicted amino acid sequences are shown below the nucleotide sequences. Nucleotide repeats used for the SSR marker are highlighted with black boxes and white letters. Primer locations are underlined, in which those of the existing mint SSRs (EMM_007 and EMM_049; Kumar *et al*. 2015) are indicated with dotted lines.

Contig28
Gene ID: augustus_masked-Contig28-abinit-gene-0.2 (homology to G-box-binding factor 4-*like*)
Upper case = putative exons (homology to *Perilla frutescens* cDNA (JZ581807))

```
     14690     14680     14670     14660     14650     14640     14630     14620     14610     14600     14590     14580
gggatggcggcggtgagtgggagcgtcgcaggCGGCGGCGGGAGAGGGAAGAGGAGGGTGGCGGTGGACGAAGTGCCGCTGGATAAGGCGACGCAGCAAAAACAGAGAAGAATGATTAAA
   M  A  A  V  S  G  S  V  A  G  G  G  G  R  G  K  R  R  V  A  V  D  E  V  P  L  D  K  A  T  Q  Q  K  Q  R  R  M  I  K

Cont028-gene0.2 F  -->
     14570     14560     14550     14540     14530     14520     14510     14500     14490     14480     14470     14460
AATAGGGAGTCTGCTGCTAGGTCCAGAGAGCGGAAGCAGgtttagcttttagcttttcttctctctctctctctctaagtatttttattgttcttgttttcccccaattcttaaaggaa
N  R  E  S  A  A  R  S  R  E  R  K  Q

                                                 <--  Cont028-gene0.2 R
     14450     14440     14430     14420     14410     14400     14390     14380     14370     14360     14350     14340
gcaactttaatcactgcttaatttttatgcttcaatttttgcttgcttctagGCTTATACCGTTGAGTTGGAGTCACTGGTGACACAACTAGAGGAGGAGAACGCTAGGCTTTTGAGAGA
                                                    A  Y  T  V  E  L  E  S  L  V  T  Q  L  E  E  E  N  A  R  L  L  R  E

     14330     14320     14310     14300     14290     14280     14270     14260     14250     14240     14230     14220
AGAGgtgcctctctctctaaagatgcactcaattgcctaaaaataacattttgtttgtatttttattctcttccaaatttgctagcaacactttacttctgtaattctatcttttatgt
 E
```

**Supplemental Fig. 3.** (continued)

Contig30

Gene ID: augustus_masked-Contig30-abinit-gene-0.5 (homology to probable alpha,alpha-trehalose-phosphate synthase [UDP-forming] 9)

Upper case = putative exons (homology to *Phaseolus acutifolius* cDNA (HO779327))

```
      19670       19660       19650       19640       19630       19620       19610       19600       19590       19580       19570       19560
GAAGCAACTGATGGATCTTCCATGGAAGTTAAAGAGAGTGCATTGGTCTGGCATCATAGGGATGCAGACCCTGACTTCGGCTCCTGCCAGGCCAAGGAACTCCTAGATCATATGGAAAAT
E  A  T  D  G  S  S  M  E  V  K  E  S  A  L  V  W  H  H  R  D  A  D  P  D  F  G  S  C  Q  A  K  E  L  L  D  H  M  E  N

                                                   Cont030-gene0.5 F  -->
      19550       19540       19530       19520       19510       19500       19490       19480       19470       19460       19450       19440
GTTCTTGCAAATGAACCCGCTGTTGTGCAAAGGGGCCAGCATATTGTTGAAGTGAAGCCACAAgtacgtctctctctctctctctctcattaatacttcaactgttgcactcatat
V  L  A  N  E  P  A  V  V  Q  R  G  Q  H  I  V  E  V  K  P  Q

                                  <--  Cont030-gene0.5 R
      19430       19420       19410       19400       19390       19380       19370       19360       19350       19340       19330       19320
cattggaataaatgtagGGTGTGACGAAAGGTTTGGCTGCGGAGAAAGTGCTCTCAATGATGGCAAACAATGGAGAGGCACCAGATTTTGTTATGTGTATCGGGGATGATAGATCAGATG
                    G  V  T  K  G  L  A  A  E  K  V  L  S  M  M  A  N  N  G  E  A  P  D  F  V  M  C  I  G  D  D  R  S  D  E

      19310       19300       19290       19280       19270       19260       19250       19240       19230       19220       19210       19200
AAGACATGTTCGAGAGCATATTAAACGCCGTATCAAATCCATCCAACACTGCAGTTCCAGAGATCTTTGCTTGCACCGTTGGGCAAAAACCGAGCAAGGCTAAATATTATCTCGATGATA
  D  M  F  E  S  I  L  N  A  V  S  N  P  S  N  T  A  V  P  E  I  F  A  C  T  V  G  Q  K  P  S  K  A  K  Y  Y  L  D  D  T

      19190       19180       19170       19160       19150       19140       19130       19120       19110       19100       19090       19080
CTTCAGACGTTCTGAGAATGCTTCGAGGTCTTGCCTCCGCTTCTAACCCGAAGCCTAGGCGGGATACCCAATTCAAGGTTGCTTTCGATAGCGTTTTCTGAcaaaaatcgaggtaggctc
  S  D  V  L  R  M  L  R  G  L  A  S  A  S  N  P  K  P  R  R  D  T  Q  F  K  V  A  F  D  S  V  F  *
```

**Supplemental Fig. 3.** (continued)

Contig39
Gene ID: maker-Contig39-augustus-gene-0.9 (homology to GTP-binding protein)
Upper case = putative exons (homology to *Mimulus guttatus* cDNA (GR129301))

```
      9100      9090      9080      9070      9060      9050      9040      9030      9020      9010      9000      8990
acagccaaaccaggaaaggATGGATCGAGAATCGCTGGAGAAGCGACCGGCGATCTTCGTCGTCGGATCCCCCAACGTCGGTAAACGCACCTTACTCTCACgtaagccctcttctctctc
                    M  D  R  E  S  L  E  K  R  P  A  I  F  V  V  G  S  P  N  V  G  K  R  T  L  L  S  R

      8980      8970      8960      8950      8940      8930      8920      8910      8900      8890      8880      8870
tctctggatcgcctgttgaagcatttctggtattttcttccgttgttttattagtaggttattgtgaagctccaggctgcagtgaggtttgaatcatggggatttagaaactttgctgtt

      8860      8850      8840      8830      8820      8810      8800      8790      8780      8770      8760      8750
cgatttagtgtaatgattaagtggcttaaccgcggattgtttaatttttactatgttttggatatttcgtgttgctaaattcttacctttgttattgaaattggcaactcaatgcttccc

      8740      8730      8720      8710      8700      8690      8680      8670      8660      8650      8640      8630
actgagctgcagataacaatgctttagaactaaggccttgagacaaaaactgaccagtgggatataattgtttggatacctcgctcaatttcatcatttacatcactcaatttcatcatt

      8620      8610      8600      8590      8580      8570      8560      8550      8540      8530      8520      8510
tacatcactcaatttcatcatttgtgcaaagagttggatttttttagttcctgttttggaaattgtttgttgaggattgtgtcttttgttctcttcctcttctgattatgcttatttggt

      8500      8490      8480      8470      8460      8450      8440      8430      8420      8410      8400      8390
ggagaatgcctcttattgtaattcctagttctagtattttattgctatgctctcccctgcctattttggaatgatgaatttggtgtagtacttctgttgtataaaaataagtttatgctt

                                                                                 Cont039gene0.9 F  -->
      8380      8370      8360      8350      8340      8330      8320      8310      8300      8290      8280      8270
ttaattatgcatagcaactgaaagataaattgttataactggatgtagGACTACTTAAGGTTGATTTTGATGATGCCTCCGACACATCTCCCGAATTATCTGTCCATGGgtaagttctga
                                                  L  L  K  V  D  F  D  D  A  S  D  T  S  P  E  L  S  V  H  G

                                                                                 <--  Cont039-gene0.9 R
      8260      8250      8240      8230      8220      8210      8200      8190      8180      8170      8160      8150
aaggctaattggctctctctctctctctctctagtagttattaagcattcaagtgttagcctgaatgtgatttcattgtcagGTGGACAATCAACACAAAGTATTATACAGCAGATGT
                                                                                 W  T  I  N  T  K  Y  Y  T  A  D  V

      8140      8130      8120      8110      8100      8090      8080      8070      8060      8050      8040      8030
TGCTGTATGGACAGCTCATCTTTCCGATGAGCTATCTATAGCAAGCTTTCCAGATGTGGACCGGATAGTTGCTCTGGTTATGGTATTCGACACCAGTGAGgtaagcctaagtccattttt
 A  V  W  T  A  H  L  S  D  E  L  S  I  A  S  F  P  D  V  D  R  I  V  A  L  V  M  V  F  D  T  S  E

      8020      8010      8000      7990      7980      7970      7960      7950      7940      7930      7920      7910
tcaccttttgctacgatgcacaaaatcctctgttctttatgtgtttaagtgaaaaatggtattttctgtgtccaaatattctctctcaatcttattagatgtatgatatgcgatttgcag

      7900      7890      7880      7870      7860      7850      7840      7830      7820      7810      7800      7790
CTCTCGACTCTAGTTGCTCTTAAAGAATGGGTATCACAGACTAACATCCAGAACTGCTATGCATGGGTAAAAAGGTTGATCTCCTTCCTAGCCATCCAGCACATGCGGAGTACAAGAGAC
L  S  T  L  V  A  L  K  E  W  V  S  Q  T  N  I  Q  N
                                                       L  L  C  M  G  K  K  V  D  L  L  P  S  H  P  A  H  A  E  Y  K  R  R

      7780      7770      7760      7750      7740      7730      7720      7710      7700      7690      7680      7670
GCTTGATGAAGCTTGGAGAATCCTCGGTCAATTCTCATCTAGACTTCTCTGATTATGGTATATCCGAGAATGAAGGAAGTAGTTTATTGGGAGATGATGAGCCATCCTTTGATTTCAAGA
  L  M  K  L  G  E  S  S  V  N  S  H  L  D  F  S  D  Y  G  I  S  E  N  E  G  S  S  L  L  G  D  D  E  P  S  F  D  F  K  R

      7660      7650      7640      7630      7620      7610      7600      7590      7580      7570      7560      7550
GATCTTGCACGGAATGGTGTCTTGAACACAACATTGAATATGTTGAAGCTTGTGCATCGAATGCTGATTTTGACCAATCTaagatcattcactacgtattttcccgtacttatgaaatgt
  S  C  T  E  W  C  L  E  H  N  I  E  Y  V  E  A  C  A  S  N  A  D  F  D  Q  S
```

**Supplemental Fig. 3.** (continued)

Contig40

Gene ID: maker-Contig40-augustus-gene-0.3 (homology to serine/threonine-protein kinase HT1-*like*)

Upper case = putative exons (homology to *Mimulus guttatus* cDNA (GO959273))

```
      8490      8480      8470      8460      8450      8440      8430      8420      8410      8400      8390      8380
aaATGGGATCTGGAAACGGGTTGTACCCGGGTGCAGAGTTGGATTTGGACCCGAAATGGTTGATTGATCCTAGGCTTCTTTTTGTTGGGCCTAAGATCGGAGAAGGAGCTCATGCCAAAG
   M  G  S  G  N  G  L  Y  P  G  A  E  L  D  L  D  P  K  W  L  I  D  P  R  L  L  F  V  G  P  K  I  G  E  G  A  H  A  K  V

      8370      8360      8350      8340      8330      8320      8310      8300      8290      8280      8270      8260
TCTACGGTAAgttgacttgttacttcttctgtctgatcttgattttccttaagtgaaaatagcttatgcgatttaagtattctgaaattatcaatgaagatagaaagctaccgctaattc
  Y  G  K

      8250      8240      8230      8220      8210      8200      8190      8180      8170      8160      8150      8140
caggaaactctgaatatttttttatgtttgaaatgggagagcagtgttgcataacaaatttagaaattttctgccatgaaaaaaataataataataaataataaaataaaataaaataac

      8130      8120      8110      8100      8090      8080      8070      8060      8050      8040      8030      8020
actgaaagttgtatttttgatgagatgtttgttcttgtggttgtggagtactttttgattgtggattgtcagctcaaattttatttttgtttggcctttttttttttgtaaagATACAAA
                                                                                                                  Y  K

                                                                                                  Cont040-gene0.3 F  -->
      8010      8000      7990      7980      7970      7960      7950      7940      7930      7920      7910      7900
AATCAGAATGTTGCTATCAAAATAATTCAGAGAGGGGAGACAGCTGAAGAAATTGCAAAGAAGGAGGGAAGATTTGCAAGAGAAGTTGCAATGCTTTCCAGAGTCCAACATAAGAATTTA
N  Q  N  V  A  I  K  I  I  Q  R  G  E  T  A  E  E  I  A  K  K  E  G  R  F  A  R  E  V  A  M  L  S  R  V  Q  H  K  N  L

      7890      7880      7870      7860      7850      7840      7830      7820      7810      7800      7790      7780
GTTAAGgttgtatatttattacaaagatgagtgatcttttgctgctgtaataataaattatggcttcaaatcacacaaatctcacatacctaatctttctgtgtgtgtgtgtgtgtgtta
V  K

                                                                                      <--  Cont040-gene0.3 R
      7770      7760      7750      7740      7730      7720      7710      7700      7690      7680      7670      7660
gtgtgatatttgtaatgagattgcttaagatggatgttttggagattagttgttagcatttcttagtgactcctttcctctttatagTTCATTGGAGCTTGCAAGGAACCTGTCATGGTT
                                                                                         F  I  G  A  C  K  E  P  V  M  V

      7650      7640      7630      7620      7610      7600      7590      7580      7570      7560      7550      7540
ATAGTGACTGAGCTTCTTCTTGGTGGGACGTTGCGGAAGATACTTAATTAACCTTCGCCCTAGTCTCTTGATAATTCGGGGTAGCTATGGCATTTGCGCTTCAGATATAGCCTGTGCAAT
I  V  T  E  L  L  L  G  G  T  L  R  K
                                       Y  L  I  N  L  R  P  S  L  L  I  I  R  G  S  Y  G  I  C  A  S  D  I  A  C  A  M

      7530      7520      7510      7500      7490      7480      7470      7460      7450      7440      7430      7420
GAgtgttgcacgcgcgagaatcatccatcggacctgaaaacctcgaatggttcttgtctttttcccacagccttagttggcgagcgagcgagaggagcgagtagcaagaggttgttagtg
```

**Supplemental Fig. 3.** (continued)

Contig55
Gene ID: maker-Contig55-augustus-gene-0.4 (homology to mitochondrial pyruvate dehydrogenase E1 component alpha)
Upper case = putative exons (homology to *M.* × *piperita* cDNA (AW255672))

```
     12710     12720     12730     12740     12750     12760     12770     12780     12790     12800     12810     12820
atcaaaaactgatattagctcatgatctagcaacTGAAAAGGAGATGAAGgtaataaaatagtctcttgcagccatttgcctcagatctatggtttggaaccattgatttggttatggt
                                   E  K  E  M  K

     12830     12840     12850     12860     12870     12880     12890     12900     12910     12920     12930     12940
agaacttttctttctgttataaacaagaatatgtggacattgcactatcaaatcaaaagtttgacgtggctctgctaaagaatatgaccatctgctgttttctttttctaagGACACTGA
                                                                                                                 D  T  E

     12950     12960     12970     12980     12990     13000     13010     13020     13030     13040     13050     13060
GAAAGAAGTTAGGAAGGAGGTGGATGACGCCATTGCAAAAGCTAAGgttagcacttgagcattatattttgcttcagatgaatccacagtgaaaggcaaattttaactaatagttcaatt
 K  E  V  R  K  E  V  D  D  A  I  A  K  A  K

     13070     13080     13090     13100     13110     13120     13130     13140     13150     13160     13170     13180
gcatttatttggttttgcagGAGAGCCCTGCGCCTGCTCCCCCCGAACTGTTTACTAATGTGTACGTCAAAGGCTTGGGAGTCGAGgtacaacttctttaaaacatcccgatattttctc
                    E  S  P  A  P  A  P  P  E  L  F  T  N  V  Y  V  K  G  L  G  V  E

     13190     13200     13210     13220     13230     13240     13250     13260     13270     13280     13290     13300
gtgaaatacatggtcttcaaacttgctcatcgctgccaatgctggtttaaccaagattgctgcttaatcatgacagtcgtctcttgttatggatatcttgaactggttacctgatattga

                                                                                 Cont055-gene0.4 F  -->
     13310     13320     13330     13340     13350     13360     13370     13380     13390     13400     13410     13420
acaaattgtcgatggttaagctataagtatttagtaatggaatgtgatttgtgtgtgattgcagGCTTTTGGAGCGGATAGGAAAGAATTGAGAGCTGTGCTTCCATGAAGGAACATTCC
                                                                 A  F  G  A  D  R  K  E  L  R  A  V  L  P  *

     13430     13440     13450     13460     13470     13480     13490     13500     13510     13520     13530     13540
ATTTCCATGTGTGGCTGCTCAGTGATAAATCAAGGCAAATAAACCAAAGCAGTATTTAGATTTTCTGTATTTGTATGTCGATTTAGTTATAACATTGTGTGTGTGTGGGGTGTGTGTTTC

<--  Cont055-gene0.4 R
     13550     13560     13570     13580     13590     13600     13610     13620
CACATTGAAATTCAGAACTTTGAGATGCTAGAACAACCATACTATTTTGCAGCAATGTAGAATTTCACTTTCCAGA
```

**Supplemental Fig. 3.** (continued)

Contig119

Gene ID: augustus_masked-Contig119-abinit-gene-0.0 (homology to transcription factor Pur-alpha 1 isoform X1)

Upper case = putative exons (homology to *Plectranthus barbatus* cDNA (JZ732539))

```
      1760      1770      1780      1790      1800      1810      1820      1830      1840      1850      1860      1870
atggaagggaattcttctgggggcggcgatagaggaggaggaggagggggtaatgatgtggaattgctttgcaaaacactgcaGGTGGAGCACAAGCTATTCTACTTTGACCTCAAGGAG
M  E  G  N  S  S  G  G  G  D  R  G  G  G  G  G  G  N  D  V  E  L  L  C  K  T  L  Q  V  E  H  K  L  F  Y  F  D  L  K  E

      1880      1890      1900      1910      1920      1930      1940      1950      1960      1970      1980      1990
AATGCCCGAGGGCGTTACCTCAAGATCTCTGAGAAGACTTCTGCCACAAGGTCAACCATCATAGTACCTTCCAACAGCATTGCCTGGTTCCTTGATCTCTTCAATTACTATGTCAATTCG
N  A  R  G  R  Y  L  K  I  S  E  K  T  S  A  T  R  S  T  I  I  V  P  S  N  S  I  A  W  F  L  D  L  F  N  Y  Y  V  N  S

      2000      2010      2020      2030      2040      2050      2060      2070      2080      2090      2100      2110
GATGACCAGGATGTCTTTAGCAAAGAACTTCAGCTCGATACCAAGgcaccctttcatatccactttctttattctatttctttattttattcatgctctaagttttctttgcctcatatc
D  D  Q  D  V  F  S  K  E  L  Q  L  D  T  K

      2120      2130      2140      2150      2160      2170      2180      2190      2200      2210      2220      2230
tgcttgcagGTTTTTTATTTTGATGTTGGAGAGAACAGAAGGGGCCGCTTTCTCAAAgttagtttctatctatctctatgatttgtcagcaattactgattctgcttattatgtatacag
         V  F  Y  F  D  V  G  E  N  R  R  G  R  F  L  K

      2240      2250      2260      2270      2280      2290      2300      2310      2320      2330      2340      2350
tagttgcattaacaaacgctataattgaacaaattatttgaactgtgaggtgttgaactctctaatatttccagttggtaatagatgtctcgaagagatgctatatgtgatttctagtgt

      2360      2370      2380      2390      2400      2410      2420      2430      2440      2450      2460      2470
tgtttctcagactcacatattgtatacgcccaataaaaactttgttatgcaatctataacatcatagtcgtcatattccaattcctattcccaatattcaagcaggcatattacgatcac

      2480      2490      2500      2510      2520      2530      2540      2550      2560      2570      2580      2590
acatccttcaatcgtttcccttgaacactttcttacaaaaattctgaacatgatttgttatattttccgtgctaaagatataattaaggagcatgagtttgtggttttgtatagcactaa

      2600      2510      2620      2630      2640      2650      2660      2670      2680      2690      2700      2710
tctatatcatttgcctcctttgggaaatcaattatctaaatcatacttgtatagtttactcactcttaggcggatcaaaatgtctgtttttttgaatgttttgttggatatgaactaaac

      2620      2730      2740      2750      2760      2770      2780      2790      2800      2810      2820      2830
atcatcactggttgctgatgtttgttgtctatctactacttatacttttaagatacatattcaacgtttttagttccccatatcttcctgattttctagaaaacagatgttaactagcgg

      2840      2850      2860      2870      2880      2890      2900      2910      2920      2930      2940      2950
tgagaattaagcccacagacgcatctcttcttcaacctcttggatgcccaatagggacgcggaggatgaggtttcattgttagtggtctgtaacgcttgttgaggcgtgctttgcaaatt

      2960      2970      2980      2990      3000      3010      3020      3030      3040      3050      3060      3070
gcttggccatgtctttatctttcctcttgcttttctgattttgaatgctttgaaaaaagtgtggcacatgtgcacagatgtgtgtcctaaatttgttgagtgatgtcgatgtgccgtagt

      3080      3090      3100      3110      3120      3130      3140      3150      3160      3170      3180      3190
gcgcatcccaagttgaggaaggaaaagtctctctttcacgcgtgttaattaaaacctatcaatgtaaagttgtcttaagtaaataagtttcatcttcaatatgtgagATATCTGAAGCTT
                                                                                                              I  S  E  A  L

            Cont119-gene0.0 F  -->
      3200      3210      3220      3230      3240      3250      3260      3270      3280      3290      3300      3310
TGTCAGCCGAAACCGTAGTACTATTATTGTTCCTGCTGGAGGTGCCGGTGATGAAGGGTGGGCAGCCTCAGAAATATTTTGGCAAAAAAGAAACTTCACGGCTAGTCATTGTCCAAATCA
 V  S  R  N  R  S  T  I  I  V  P  A  G  G  A  G  D  E  G  W  A  A  S  E  I  F  W  Q  K  R  N  F  T  A  S  H  C  P  N  Q

                                                                                        <--  Cont119-gene0.0 R
      3320      3330      3340      3350      3360      3370      3380      3390      3400      3410      3420      3430
GgtatatggtgtaatttttttattatctagccactcgttattgaaaacaaaagcttatgtgactttggttgtgtgtgtgtgtaagCATCAGCAGAACTCTGAAGCCCCAGAACGCCTTGT
                                                                                       H  Q  Q  N  S  E  A  P  E  R  L  V

      3440      3450      3460      3470      3480      3490      3500      3510      3520      3530      3540      3550
AGGGCTTTCAGATGATTGTGGGGAGTCTGGTTgtgtggtgcaagtcatagtgtgtgaaggtctgccctcagcgtcctgggcgtgagcgtgaatggttgatcctcagcagcgttggtagtg
 G  L  S  D  D  C  G  E  S  G
```

**Supplemental Fig. 3.** (continued)

Contig130
Gene ID: maker-Contig130-augustus-gene-0.5 (homology to TATA-box-binding protein 2-*like* isoform X1)
Upper case = putative exons (homology to *Ocimum basilicum* cDNA (DY334650))

```
      7410      7420      7430      7440      7450      7460      7470      7480      7490      7500      7510      7520
ttatcccaggggttgtgttaatttacatctctagttgatactgaatttgtgatgcttccagCGTTTTGCTGCGGTGATTATGAGGATTCGAGAACCAAAAACAACAGCGCTAATATTTGC
                                                              R  F  A  A  V  I  M  R  I  R  E  P  K  T  T  A  L  I  F  A

      7530      7540      7550      7560      7570      7580      7590      7600      7610      7620      7630      7640
ATCTGGAAAGATGgtaagtagagatgtacctcttgaaatcttgtgcaactttttgaattttgccaggtgaaaggtttgtggtacttccatttcggcaccatttgggtgcttctccttatc
 S  G  K  M

      7650      7660      7670      7680      7690      7700      7710      7720      7730      7740      7750      7760
cattgtttagattttcacgtctgttcgtagtaatggcctaatgggtatttgccagtctggactcgatattcttgttttccatatatgctaatttttttcttcatgttttcagGTGTGTAC
                                                                                                                 V  C  T

      7770      7780      7790      7800      7810      7820      7830      7840      7850      7860      7870      7880
AGGAGCCAAGAGCGAACAGCAATCCAAATTGGCTGCTCGTAAGgtatagcatatcatttgatgaaaatgtttggtttgcaaaattataactggggagtgtctgaaaaaaagcatcaccaa
 G  A  K  S  E  Q  Q  S  K  L  A  A  R  K

      7890      7900      7910      7920      7930      7940      7950      7960      7970      7980      7990      8000
ccctttgatcaaaccaaaatgtttgataagctctttaggtttattgacctggaaatattaagtaaattcatctagggaattcatctaggaaattcatctatctcatgttcaagtgattat

      8010      8020      8030      8040      8050      8060      8070      8080      8090      8100      8110      8120
ctttccttcctctcactttggatggctcctaactatttatttcagTATGCTAGAATTATTCAGAAGCTTGGATTCCCTGCAAAATTCAAGgtaccattctatattctcataggcaattga
                                              Y  A  R  I  I  Q  K  L  G  F  P  A  K  F  K

      8130     82140     82150     82160     82170     82180     82190      8200      8210      8220      8230      8240
ctctaatgacatcttatagtatttttttcaatattaaatctagagatttatcctcaattttcttcctttcccatctcatagGATTTCAAGATTCAGAACATTGTTGGCTCATGTGATGTA
                                                                                  D  F  K  I  Q  N  I  V  G  S  C  D  V

      Cont130-gene0.5 F  -->
      8250      8260      8270      8280      8290      8300      8310      8320      8330      8340      8350      8360
AAATTTCCCATTCGACTTGAAGGGCTGGCCGCTTTTTCCAGTGTGAGTGGTTCATTTGCCTGTGgttttattatgtccttgtatttgtgttatctgaaaaaatcatgctaaaagaaaaga
K  F  P  I  R  L  E  G  L  A  A  F  S  S  V  S  G  S  F  A  C

      8370      8380      8390      8400      8410      8420      8430      8440      8450      8460      8470      8480
ataggggtgctaacggaaatctgaaaattccattctattgttgttatatatatattatatatatatatatatatagatagatagatagatagatatatgcatttattgctttcacttgaa

                                                                        <--  Cont130-gene0.5 R
      8490      8500      8510      8520      8530      8540      8550      8560      8570      8580      8590      8600
gtggacagcaatttcttgagttaccgcgttcgaagtaccttcattgtgtttatcgtatcttttgttgcttcagTATGAGCCGGAATTGTTCCCTGGATTAATCTACCGGATGAAACAACC
                                                                          Y  E  P  E  L  F  P  G  L  I  Y  R  M  K  Q  P

      8610      8620      8630      8640      8650      8660      8670      8680      8690      8700      8710      8720
TAAAATTGTGCTCCTTATATTTGTCTCTGGGAAGATTGTTCTTACTGGAGCTAAGgtaaataattgcaacttgccatttttccaggtcaccatcttcctagtaatctgcttataatctgg
 K  I  V  L  L  I  F  V  S  G  K  I  V  L  T  G  A  K

      8730      8740      8750      8760      8770      8780      8790      8800      8810      8820      8830      8840
tgatgctgatatataccattttctgttataacctttttcagGTTAGAGATGAAACATATACTGCCTTTGAGAACATATACCCTGTTCTCACAGAATTTCGGAAAGTTCAGCAATGgtatg
                                          V  R  D  E  T  Y  T  A  F  E  N  I  Y  P  V  L  T  E  F  R  K  V  Q  Q  W
```

**Supplemental Fig. 3.** (continued)

Contig138

Gene ID: maker-Contig138-augustus-gene-0.5 (homology to agamous-*like* MADS-box protein AGL8)

Upper case = putative exons (homology to *Mimulus guttatus* cDNA (GR114416))

```
     12990     12980     12970     12960     12950     12940     12930     12920     12910     12900     12890     12880
ttcacccttcacaattaactctttcttcttgaaatttaatgttgcaagttaaaatcatgcgactgtgcttctactgttttgatatgacagTATGGGAAGAATTCTTGAACAATACGAGCA
                                                                                           Y  G  K  N  S  *  T  I  R  A 

            Cont138-gene0.5 F  -->
     12870     12860     12850     12840     12830     12820     12810     12800     12790     12780     12770     12760
ACAATCCTACTCTCACAAACAAATCGGCACTTCTTATCAACAAAAGgtgcttcaagctagcttcctagaatatatgtgtgtgtgtgtgtgtgagtttttttttctttttttcttttat
T  I  L  L  S  Q  T  N  R  H  F  L  S  T  K  G

                                                                <--  Cont138-gene0.5 R
     12750     12740     12730     12720     12710     12700     12690     12680     12670     12660     12650     12640
aaatttaatttaatgattatctgaatgaattacttgctgtatatggtacggttgtgatgtagGAAAACTTGTTCCTAGACTACCCAAAACTCGTGAGTCGTCTTCAACTTTTGCAGAGAA
                                                                 K  L  V  P  R  L  P  K  T  R  E  S  S  S  T  F  A  E  N 

     12630     12620     12610     12600     12590     12580     12570     12560     12550     12540     12530     12520
CTATGAGgtactattttctagcatttgtatatgatcatattctaagaaatggcatatatgtatgtttaattatttataaatataaaattccattttgcatacataattcaagggtttggc
 Y  E
```

**Supplemental Fig. 3.** (continued)

Contig171
Gene ID: maker-Contig171-augustus-gene-0.6 (uncharacterized protein)
Upper case = putative exons (homology to *Salvia miltiorrhiza* cDNA (CV168427))

```
       7490       7480       7470       7460       7450       7440       7430       7420       7410       7400       7390       7380
tagatatccgtgtttccatttgattgttctcataagaagaactggatgcactcttttttgCAGCTGATCGTCTTGATACATTTTTGAAGCAATTTCCTGAATTTCCAAATGCTTTTTTAAT
                                                             A  D  R  L  D  T  F  L  K  Q  F  P  E  F  P  N  A  F  L  I

       7370       7360       7350       7340       7330       7320       7310       7300       7290       7280       7270       7260
TGGAGGTGCACCAGACATATTTGTTACTGAACTAGCTGATCAGgtacgtgataacatcataagatactacgggtctgattctttcggtcttattagttgttgctagtttttctttacaga
 G  G  A  P  D  I  F  V  T  E  L  A  D  Q

                                                                                                                 Cont171-gene0.6 F  -->
       7250       7240       7230       7220       7210       7200       7190       7180       7170       7160       7150       7140
ctgtcatccataaaaactctccttctctatcttatattgaaatttattggtctcatatgttgtttgaccattctgtaaccagCTTCAGGAATTGAAGGTAGAGCCTGTGCTTCTTCATTA
                                                                                    L  Q  E  L  K  V  E  P  V  L  L  H  Y

                                                                                                              <--  Cont171-gene0.6 R
       7130       7120       7110       7100       7090       7080       7070       7060       7050       7040       7030       7020
CTTATCTCATATAAAAGTTATTCAAGgtactctctctctctctctctcacacacacacacacacagacacaactggtttgatgtctttaatacctgtacagGCCTAGAACTAAGAGTGGC
 L  S  H  I  K  V  I  Q  G                                                                             L  E  L  R  V  A

       7010       7000       6990       6980       6970       6960       6950       6940       6930       6920       6910       6900
AACAAGTACAAGACTAAAAGCATGTCTATATAGCTTCACATCTCCTGGTGGTCCTATGTACCCAACAAGAGCTGTTCGTCACGCGGCGTGGGATGAATTAAATGATCTTTTCCCTgtaag
 T  S  T  R  L  K  A  C  L  Y  S  F  T  S  P  G  G  P  M  Y  P  T  R  A  V  R  H  A  A  W  D  E  L  N  D  L  F  P  V  R

       6890       6880       6870       6860       6850       6840       6830       6820       6810       6800       6790       6780
gcattacattttccatgtttactatgataactttgatactgcatgtgtttcttctcttaagataccagttttggacacatgcatgcaaccttttttattcaacctagtagtacctggacat
 H  Y  I  F  H  V  Y  Y  D  N  F  D  T  A  C  V  S  S  L  K  I  P  V  L  D  T  C  M  Q  P  F  Y  S  T  *
```

**Supplemental Fig. 3.** (continued)

Contig23404
Gene ID: maker-Contig23404-augustus-gene-0.7 (homology to early light-induced protein 1, chloroplastic isoform)
Upper case = putative exon (homology to *M.* × *piperita* cDNA (AW255005))

```
                                        (EMM_007 F)  -->            EMM_007-Mp F  -->
    18050     18040     18030     18020     18010     18000     17990     17980     17970     17960     17950     17940
tatatatatttcacttttccatgcattcattactccaccaaccattacTAACAATCTCCACTTCTTTGGAAAACCAGCCTTTGCAATAATACATTGTTTCACCAGCTCTCTCTCTCTTCA

                                    <--  (EMM_007 R)            <--  EMM_007-Mp R
    17930     17920     17910     17900     17890     17880     17870     17860     17850     17840     17830     17820
TTTTCTTCTTCTTCTTCATCTCTCTCTCTCTCTCACACACACACACACACACACACACTTAAGTAAGTGAAATGGCTGCATCATGCTATGGAATGTCTGCAATTTCATGTGGGAGCCC
                                                                         M  A  A  S  C  Y  G  M  S  A  I  S  C  G  S  P

    17810     17800     17790     17780     17770     17760     17750     17740     17730     17720     17710     17700
GGTTGCTAGAAGGGGTGGCATGACTCAGCTTCTCGGAGCTTCTAAGTTTGCTCTTCCGTTGAAGAGAGATGTCAAGTTTAgagtgagaagcacagctgaggtattacttctcatttcttc
 V  A  R  R  G  G  M  T  Q  L  L  G  A  S  K  F  A  L  P  L  K  R  D  V  K  F  R  V  R  S  T  A  E
```

**Supplemental Fig. 3.** (continued)

Contig54312
Gene ID: maker-Contig54312-augustus-gene-0.4 (homology to sucrose-phosphatase)
Upper case = putative exon (homology to *M.* × *piperita* cDNA (AW255910))

```
                                                           EMM_049-Mp F (EMM_049 F)  -->
      3210      3220      3230      3240      3250      3260      3270      3280      3290      3300      3310      3320
atcaggatgtgacagtggccgatggcttaacttgggtgcatgttcatcaaacatggttgaagGAAGCAGGCCCAACCACCGACGCTGAGTGGTTATTCTAAACTCTCTCCTCCAAATAAA
  Q  D  V  T  V  A  D  G  L  T  W  V  H  V  H  Q  T  W  L  K  E  A  G  P  T  T  D  A  E  W  L  F  *

              <--  EMM_049-Mp R                       <--  (EMM_049 R)
      3330      3340      3350      3360      3370      3380      3390      3400      3410      3420      3430      3440
TACAAAAGCTAGACGAGTATTCAGAACTGTTGTTAATTTCTGCTGATGTTGATGATTCGAGTCATGTGAGGATGGAGTAACACACACAGCTCCTACCTCATAACATTTGTCTCTTATATT

      3450      3460      3470      3480      3490      3500      3510      3520      3530      3540      3550      3560
GCGAATATAAATATATACTACTAAATAATTACTTACATGCAGCTGTTGCTAATTTTTGCTGTATGTGACACGTGTGTGTatgtgcatgtatctttcttcagtcttaagagttgcgatagt
```

**Supplemental Fig. 3.** (continued)

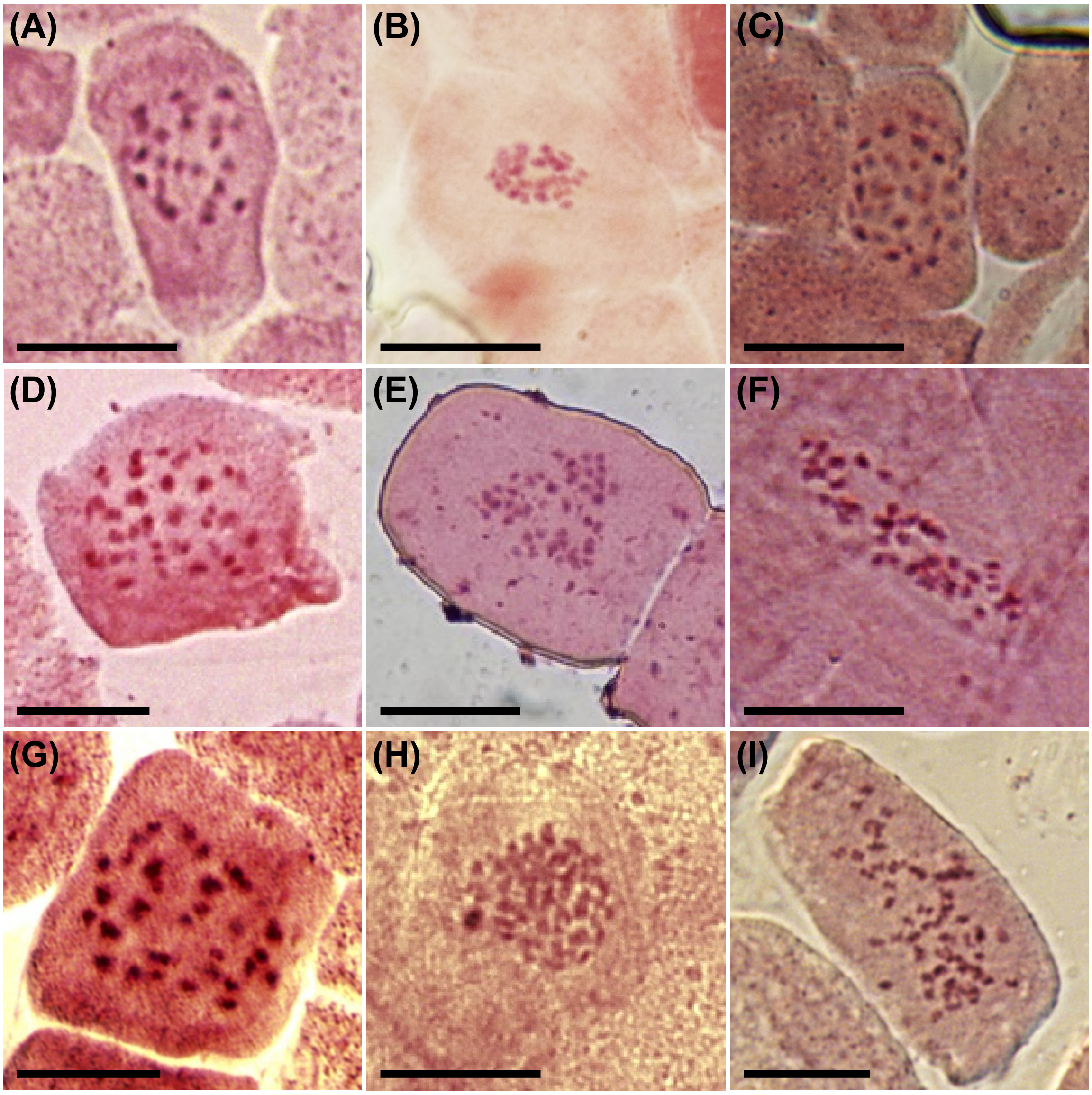


**Supplemental Fig. 4.** Photomicrographs of nine representative *Mentha* lines. (A) Apple mint [Pot] ($2n = 2x = 24$), (B) Horse mint-PI557757 ($2n = 2x = 24$), (C) Spearmint [Pot] ($2n = 4x = 48$), (D) #056 ($2n = 4x = 48$), (E) #114 ($2n = 4x = 48$), (F) #135 ($2n = 4x = 48$), (G) Kentucky colonel [Kur] ($2n = 4x = 48$), (H) Ryokubi-JP176285 ($2n = 8x = 96$), and (I) #128A ($2n = 8x = 96$). Scale bar: 10 µm.

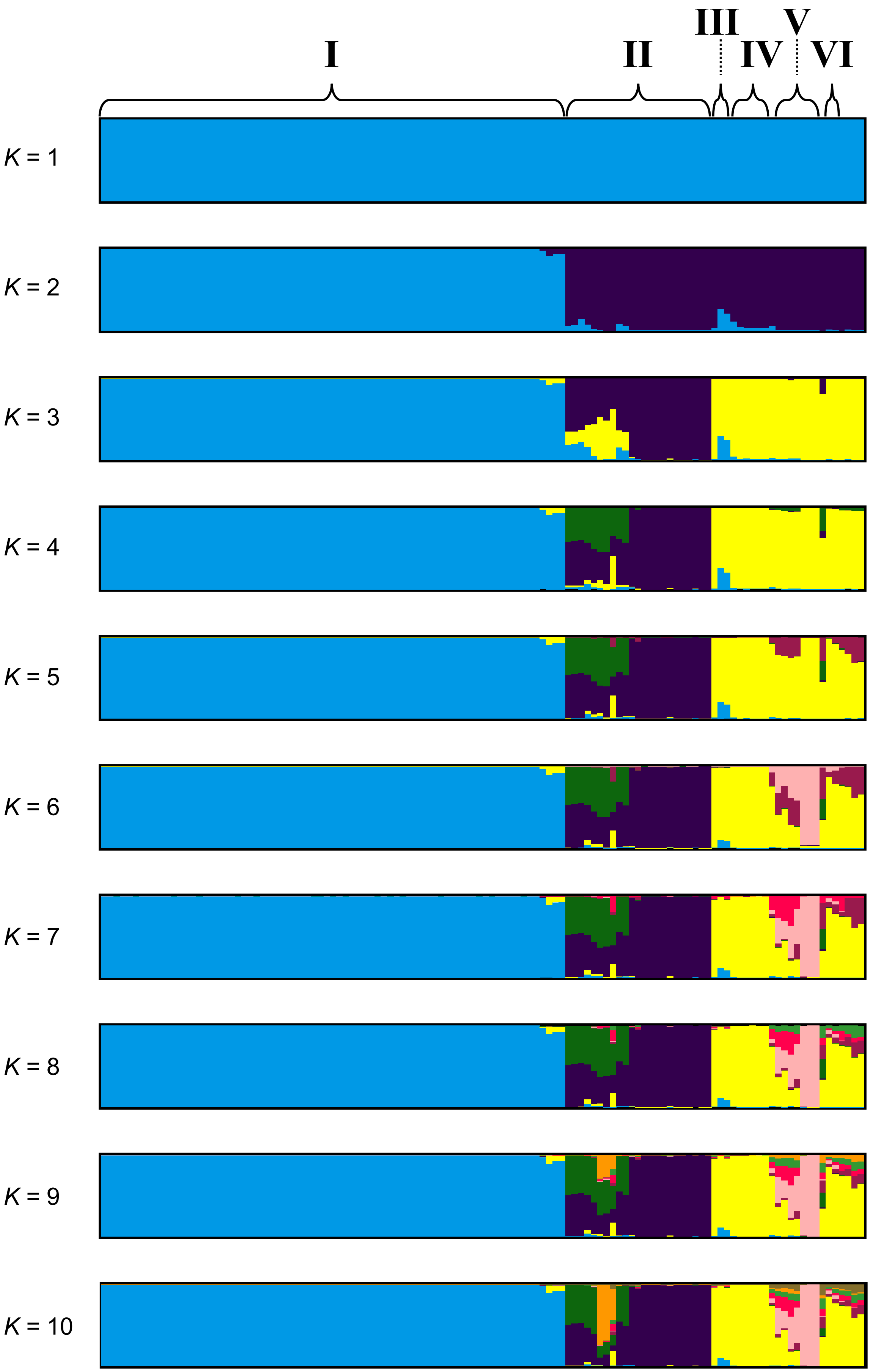


**Supplemental Fig. 5.** Genetic structures of the 120 lines based on 12 SSR markers. Results from 10 different number of subpopulations (*K* = 1-10) are shown. The colors of the barplots correspond to the assumed clusters at each *K*. The six potential groups (I-VI) observed in Fig. 2 are indicated at the top.
