# Supplementary material for: Classification of Southeast Asian mints (Mentha spp.) based on simple sequence repeat markers
Source: Breed Sci. 2022 Mar 9;72(2):181–7. doi: 10.1270/jsbbs.21058 (PMC9522532; doi:10.1270/jsbbs.21058)
Supplement: Supplementary file 2 — Supplemental Tables [file 72_181_s2.pdf]

Supplemental Table 1. List of samples used in this study

Category	Group[a]	Species[b]	Line name[c]	Information of collection[d]								Accession and code no.[e]	Sample source (field collection, genebank and seed company) and note
				Local name	Commune	District	Province or Prefecture	Country	Latitude	Longitude	Date		
Mint collected in Southeast Asian countries and Japan													
	Group I	*Mentha* sp.	#015	Lv1			Vientiane	Laos	17°58'27.0"N	102°36'28.7"E	2016.3.8		Collection in this study
	Group I	*Mentha* sp.	#017	Lv3			Vientiane	Laos	17°58'19.3"N	102°38'26.8"E	2016.3.8		Collection in this study
	Group I	*Mentha* sp.	#018	Lv4			Vientiane	Laos	17°58'19.3"N	102°38'26.8"E	2016.3.8		Collection in this study
	Group I	*Mentha* sp.	#013	Mt1			Tachileik	Myanmar	20°27'11.2"N	99°53'54.0"E	2016.3.6		Collection in this study
	Group I	*Mentha* sp.	#012	Saranae		Chatuchak	Bangkok	Thailand	13°47'50.6"N	100°32'52.6"E	2015.9.28		Collection in this study
	Group I	*Mentha* sp.	#021	Tb4			Bangkok	Thailand	13°46'30.5"N	100°29'05.1"E	2016.4		Collection in this study
	Group I	*Mentha* sp.	#061	Saranae	67 Market	Bang Mot	Bangkok	Thailand	13°39'06.1"N	100°28'47.6"E	2016.7.2		Collection in this study
	Group I	*Mentha* sp.	#062	Saranae	KMUTT group	Bang Mot	Bangkok	Thailand	13°39'06.1"N	100°28'47.6"E	2016.7.2		Collection in this study
	Group I	*Mentha* sp.	#063	Saranae	Bon Marche Market	Bang Sue	Bangkok	Thailand					Collection in this study
	Group I	*Mentha* sp.	#064	Saranae	MUPY group	Taweewattana	Bangkok	Thailand	13°47'14.7"N	100°20'10.3"E	2016.7.3		Collection in this study
	Group I	*Mentha* sp.	#065	Saranae	MUPY group	Bangkae	Bangkok	Thailand	13°42'35.8"N	100°24'34.3"E	2016.7.3		Collection in this study
	Group I	*Mentha* sp.	#140	Saranae		Hat Yai	Songkhla	Thailand	7°0'32.0"N	100°31'40.0"E	2017.6.27		Collection in this study
	Group I	*Mentha* sp.	#142	Saranae		In Buri	Sing Buri	Thailand	13°39'49.8"N	100°26'25.6"E	2017.6.30		Collection in this study
	Group I	*Mentha* sp.	#143	Saranae		In Buri	Sing Buri	Thailand	13°39'49.8"N	100°26'25.6"E	2017.6.30		Collection in this study
	Group I	*Mentha* sp.	#033	Hòm Cản Cằm	Chiềng Khoi	Yên Châu	Sơn La	Vietnam	21°01'03.1"N	104°19'26.9"E	2016.6.5		Collection in this study
	Group I	*Mentha* sp.	#037	Rau Húng		Tủa Chùa	Điện Biên	Vietnam	21°51'22.0"N	103°20'27.1"E	2016.6.6		Collection in this study
	Group I	*Mentha* sp.	#039	Rau Húng		Mường Lay	Điện Biên	Vietnam	22°02'55.7"N	103°09'49.1"E	2016.6.7		Collection in this study
	Group I	*Mentha* sp.	#040	Rau Húng	TP Điện Biên	Mường Lay	Điện Biên	Vietnam	22°02'55.8"N	103°09'49.2"E	2016.6.7		Collection in this study
	Group I	*Mentha* sp.	#043	Hòm Kim		Mường Tè	Lai Châu	Vietnam	22°22'31.8"N	102°48'45.7"E	2016.6.7		Collection in this study
	Group I	*Mentha* sp.	#048	Rau Húng		Mù Căng Chải	Yên Bái	Vietnam	21°46'59.7"N	104°09'08.6"E	2016.6.8		Collection in this study
	Group I	*Mentha* sp.	#051	Medan	Xuân Sơn	Tân Sơn	Phú Thọ	Vietnam	21°07'38.2"N	104°55'42.1"E	2016.6.9		Collection in this study
	Group I	*Mentha* sp.	#056	Rau Húng		Tam Đảo	Vĩnh Phúc	Vietnam	21°27'58.9"N	105°37'56.4"E	2016.6.10		Collection in this study
	Group I	*Mentha* sp.	#057	Quan Sơi	Đại Đình	Tam Đảo	Vĩnh Phúc	Vietnam	21°27'42.3"N	105°35'03.2"E	2016.6.10		Collection in this study
	Group I	*Mentha* sp.	#058	Rau Húng		TP Hà Nội	Hà Nội	Vietnam	21°01'43.7"N	105°50'07.9"E	2016.6.10		Collection in this study
	Group I	*Mentha* sp.	#059	Rau Húng		TP Hà Nội	Hà Nội	Vietnam	21°01'43.7"N	105°50'07.9"E	2016.6.10		Collection in this study
	Group I	*Mentha* sp.	#066	Rau Húng Chuối		Xã Binh Loc, Định Quán	Đồng Nai	Vietnam	11°11'31.5"N	107°20'57.6"E	2016.9.28		Collection in this study
	Group I	*Mentha* sp.	#071	Rau Húng	Da Lat	Xã Đăk Ơ, Bù Gia Mập	Bình Phước	Vietnam	12°02'39.5"N	107°05'22.2"E	2016.9.29		Collection in this study
	Group I	*Mentha* sp.	#072	Rau Húng		Xã Tân Tiến, Bù Đốp	Bình Phước	Vietnam	11°55'56.0"N	106°44'32.5"E	2016.9.29		Collection in this study
	Group I	*Mentha* sp.	#074	Rau Húng Chuối		Xã Tân Thành Hòn Quán	Bình Phước	Vietnam	11°30'58.1"N	106°48'17.6"E	2016.9.29		Collection in this study
	Group I	*Mentha* sp.	#078	Rau Húng Chuối		Quận Tân Bình	Thành phố phố Hồ Chí Minh	Vietnam	10°47'41.6"N	106°39'48.0"E	2016.9.30		Collection in this study
	Group I	*Mentha* sp.	#083	Rau Húng		Xã Cỗ Lũng, Huyện Phú Lương	Thái Nguyên	Vietnam	21°38'55.5"N	105°45'12.0"E	2016.11.12		Collection in this study
	Group I	*Mentha* sp.	#087	Rau Húng		Xã Thành Long, Huyện Hàm Yên	Tuyên Quang	Vietnam	21°59'28.9"N	105°04'10.7"E	2016.11.12		Collection in this study
	Group I	*Mentha* sp.	#088	Rau Húng		Xã Thành Long, Huyện Hàm Yên	Tuyên Quang	Vietnam	21°59'28.9"N	105°04'10.7"E	2016.11.12		Collection in this study
	Group I	*Mentha* sp.	#089	Rau Húng		T.T. Vĩnh Lộc, Huyện Chiêm Hóa	Tuyên Quang	Vietnam	22°08'30.5"N	105°16'24.9"E	2016.11.12		Collection in this study
	Group I	*Mentha* sp.	#091	Rau Húng		T.T. Vinh Quang, Hoàng Su Phì	Hà Giang	Vietnam	21°36'05.0"N	105°52'06.1"E	2016.11.13		Collection in this study
	Group I	*Mentha* sp.	#093	Rau Húng		Xã Hồ Thầu, Hoàng Su Phì	Hà Giang	Vietnam	22°22'04.0"N	103°34'31.2"E	2016.11.13		Collection in this study
	Group I	*Mentha* sp.	#094	Rau Húng		Xã Hồ Thầu, Hoàng Su Phì	Hà Giang	Vietnam	22°22'04.0"N	103°34'31.2"E	2016.11.13		Collection in this study
	Group I	*Mentha* sp.	#095	Rau Húng		Đường Không Tên, T.T.Tam Sơn, Quản Bạ	Hà Giang	Vietnam	23°01'06.5"N	104°58'31.4"E	2016.11.14		Collection in this study
	Group I	*Mentha* sp.	#097	Rau Húng	Thôn Sáng Phàng	Đông Hà, Quản Bạ	Hà Giang	Vietnam	23°02'37.9"N	105°00'55.4"E	2016.11.14		Collection in this study
	Group I	*Mentha* sp.	#098	Rau Húng	Xóm Cổng Trà	Xã Phố Là, Huyện Đồng Văn	Hà Giang	Vietnam	23°15'39.5"N	105°10'05.7"E	2016.11.14		Collection in this study
	Group I	*Mentha* sp.	#099	Rau Húng		Xã Lũng Cú, Huyện Đồng Văn	Hà Giang	Vietnam	23°22'01.7"N	105°19'31.4"E	2016.11.14		Collection in this study
	Group I	*Mentha* sp.	#101	Rau Húng		Xom Thien Hung, Đồng Văn, Huyện Đồng Văn	Hà Giang	Vietnam	23°15'59.6"N	105°22'04.0"E	2016.11.15		Collection in this study
	Group I	*Mentha* sp.	#102	Rau Húng		Xóm Sảng Pả A, Huyện Mèo Vạc	Hà Giang	Vietnam	23°09'12.1"N	105°23'46.2"E	2016.11.15		Collection in this study
	Group I	*Mentha* sp.	#103	Rau Húng		Xã Tát Ngà, Huyện Mèo Vạc	Hà Giang	Vietnam	23°06'10.8"N	105°25'04.8"E	2016.11.15		Collection in this study
	Group I	*Mentha* sp.	#106	Rau Húng		T.T. Tĩnh Túc, Nguyên Bình	Cao Bằng	Vietnam	22°38'38.9"N	105°52'53.4"E	2016.11.16		Collection in this study
	Group I	*Mentha* sp.	#107	Rau Húng		Xã Bành Trạch, Ba Bể	Bắc Kạn	Vietnam	22°30'08.7"N	105°48'01.7"E	2016.11.16		Collection in this study
	Group I	*Mentha* sp.	#108	Rau Húng		Ba Bể National Park, Ba Bể	Bắc Kạn	Vietnam	22°24'40.8"N	105°36'18.8"E	2016.11.16		Collection in this study
	Group I	*Mentha* sp.	#109	Rau Húng		Phủ Thông, Bạch Thông	Bắc Kạn	Vietnam	22°16'20.8"N	105°52'48.0"E	2016.11.16		Collection in this study
	Group I	*Mentha* sp.	#110	Rau Húng		T.H. Lang Son	Lạng Sơn	Vietnam	21°02'19.9"N	105°51'04.6"E	2016.11.17		Collection in this study
	Group I	*Mentha* sp.	#111	Rau Húng		Thanh Lòa, Cao Lộc	Lạng Sơn	Vietnam	21°57'20.5"N	106°50'16.1"E	2016.11.17		Collection in this study
	Group I	*Mentha* sp.	#112	Rau Húng	Còn Phạc	Còn Phạc, Cao Lộc	Lạng Sơn	Vietnam	21°58'16.0"N	106°47'49.9"E	2016.11.17		Collection in this study
	Group I	*Mentha* sp.	#114	Rau Húng		T.T. Bố Hạ, Huyện Yên Thế	Bắc Giang	Vietnam	21°26'40.4"N	106°11'56.8"E	2016.11.17		Collection in this study
	Group I	*Mentha* sp.	#115	Rau Húng	Khu 2 Quan Hóa	Quan Hóa	Thanh Hóa	Vietnam	20°23'03.0"N	105°05'59.0"E	2017.5.20		Collection in this study
	Group I	*Mentha* sp.	#119	Rau Húng	Công An	Mường Lát	Thanh Hóa	Vietnam	20°26'59.0"N	104°46'15.0"E	2017.5.20		Collection in this study
	Group I	*Mentha* sp.	#120	Rau Húng	Bản Hiết, Sơn Thủy	Quan Sơn	Thanh Hóa	Vietnam	20°20'21.0"N	104°45'51.0"E	2017.5.20		Collection in this study
	Group I	*Mentha* sp.	#121	Rau Húng	Bản Chiềng, Mường Mìn	Quan Sơn	Thanh Hóa	Vietnam	20°16'33.0"N	104°45'16.0"E	2017.5.20		Collection in this study
	Group I	*Mentha* sp.	#122	Rau Húng	Phố Cống	Ngọc Lặc	Thanh Hóa	Vietnam	20°04'55.7"N	105°22'45.4"E	2017.5.21		Collection in this study
	Group I	*Mentha* sp.	#123	Rau Húng	Hồ Chí Minh, Thượng Ninh	Như Xuân	Thanh Hóa	Vietnam	19°42'22.0"N	105°26'31.0"E	2017.5.21		Collection in this study
	Group I	*Mentha* sp.	#124	Rau Húng	Làng Tra	Nghĩa Đàn	Nghệ An	Vietnam	19°25'28.0"N	105°26'47.0"E	2017.5.21		Collection in this study
	Group I	*Mentha* sp.	#129	Rau Húng	Xa Nga My	Tương Dương	Nghệ An	Vietnam	19°16'45.2"N	104°45'33.8"E	2017.5.21		Collection in this study
	Group I	*Mentha* sp.	#130	Rau Húng	Tà Cạ	Kỳ Sơn	Nghệ An	Vietnam	19°24'49.0"N	104°04'19.0"E	2017.5.22		Collection in this study
	Group I	*Mentha* sp.	#131	Rau Húng	Mường Típ	Kỳ Sơn	Nghệ An	Vietnam	19°24'21.0"N	104°01'04.0"E	2017.5.22		Collection in this study
	Group I	*Mentha* sp.	#132	Rau Húng	Nậm Càn	Kỳ Sơn	Nghệ An	Vietnam	19°28'05.0"N	104°05'16.0"E	2017.5.22		Collection in this study
	Group I	*Mentha* sp.	#134	Rau Húng	Bảo Nam (Trung Tâm)	Kỳ Sơn	Nghệ An	Vietnam	19°24'19.0"N	104°15'56.0"E	2017.5.22		Collection in this study

Group I	*Mentha* sp.	#135	Rau Húng	Huổi Luông, Bảo Thắng	Kỳ Sơn	Nghệ An	Vietnam	19°23'39.0"N	104°23'54.0"E	2017.5.22		Collection in this study
Group I	*Mentha* sp.	#136	Rau Húng	Minh Thành, Lưỡng Minh	Tương Dương	Nghệ An	Vietnam	19°21'16.0"N	104°23'20.0"E	2017.5.22		Collection in this study
Group I	*Mentha* sp.	#137	Rau Húng	Chăm Phong, Lưỡng Minh	Tương Dương	Nghệ An	Vietnam	19°21'42.0"N	104°21'59.0"E	2017.5.22		Collection in this study
Group II	*Mentha* sp.	#117	Rau Húng	Bản Ngổ, Mường Chanh	Mường Lát	Thanh Hóa	Laos	20°24'39.0"N	104°27'55.0"E	2017.5.20		Collection in this study
Group II	*Mentha* sp.	#118		Bản Lách, Mường Chanh	Mường Lát	Thanh Hóa	Laos	20°23'43.0"N	104°28'25.0"E	2017.5.20		Collection in this study
Group II	*Mentha* sp.	#007				Hà Nội	Vietnam	21°01'32.4"N	105°50'01.3"E	2015.5.25		Collection in this study
Group II	*Mentha* sp.	#008				Hà Nội	Vietnam	21°01'32.4"N	105°50'01.3"E	2015.5.25		Collection in this study
Group II	*Mentha* sp.	#026	Rau Húng		Kỳ Sơn	Hòa Bình	Vietnam	19°23'18.8"N	104°10'56.2"E	2016.6.4		Collection in this study
Group II	*Mentha* sp.	#029	Im Kim		Mai Châu	Hòa Bình	Vietnam	20°39'34.9"N	105°05'29.8"E	2016.6.5		Collection in this study
Group II	*Mentha* sp.	#034	Hòm Cản Cằm		TP Sơn La	Sơn La	Vietnam	21°19'43.6"N	103°54'20.7"E	2016.6.6		Collection in this study
Group II	*Mentha* sp.	#035	Hòm Cản Cằm		TP Sơn La	Sơn La	Vietnam	21°19'43.6"N	103°54'20.7"E	2016.6.6		Collection in this study
Group II	*Mentha* sp.	#050	Rau Húng		Nghĩa Lộ	Yên Bái	Vietnam	21°36'08.0"N	104°30'24.6"E	2016.6.9		Collection in this study
Group II	*Mentha* sp.	#055	Quat Bán Hoán	Đạo Thù	Tam Đảo	Vĩnh Phúc	Vietnam	21°27'58.9"N	105°37'56.4"E	2016.6.10		Collection in this study
Group II	*Mentha* sp.	#073			Xã Tân Tiến, Bù Đốp	Bình Phước	Vietnam	11°55'56.0"N	106°44'32.5"E	2016.9.29		Collection in this study
Group II	*Mentha* sp.	#084	Rau Húng		Xã Na Mao, Huyện Đại Từ	Thái Nguyên	Vietnam	21°41'41.4"N	105°32'34.9"E	2016.11.12		Collection in this study
Group II	*Mentha* sp.	#113	Rau Húng Đỏ		T.T. Bố Hạ, Huyện Yên Thế	Bắc Giang	Vietnam	21°27'00.6"N	106°11'54.1"E	2016.11.17		Collection in this study
Group II	*Mentha* sp.	#125	Rau Húng	Làng Tra	Nghĩa Đàn	Nghệ An	Vietnam	19°25'28.0"N	105°26'47.0"E	2017.5.21		Collection in this study
Group II	*Mentha* sp.	#126	Rau Húng	Chơ Tân Kỳ	Tân Kỳ	Nghệ An	Vietnam	19°16'05.0"N	105°26'47.0"E	2017.5.21		Collection in this study
Group II	*Mentha* sp.	#128A	Rau Húng	Xa Nga My	Tương Dương	Nghệ An	Vietnam	19°16'44.0"N	104°45'35.2"E	2017.5.21		Collection in this study
Group II	*Mentha* sp.	#128B	Rau Húng	Xa Nga My	Tương Dương	Nghệ An	Vietnam	19°16'44.0"N	104°45'35.2"E	2017.5.21		Collection in this study
Group II	*Mentha* sp.	#133	Rau Húng	Hữu Kiệm	Kỳ Sơn	Nghệ An	Vietnam	19°22'21.0"N	104°13'12.0"E	2017.5.22		Collection in this study
Group II	*Mentha* sp.	#138	Rau Húng	Chơ Anh Sơn	Tương Dương	Nghệ An	Vietnam	18°55'56.0"N	105°04'55.0"E	2017.5.23		Collection in this study
Spearmint	*Mentha* sp.	#003		Open field		Aichi	Japan			2015.5.1		Collection in this study
Apple mint	*M. suaveolens*	Apple mint [KPU]		University Farm, Faculty of Life and Enviromental Sciences, Kyoto Prefectural University	Soraku-gun	Kyoto	Japan	34°46'30.6"N	135°45'38.3"E	2017.3.24		Collection in this study
Germplasm of public section												
Group I	*M. spicata* *	Kentucky colonel-PI637833				Oregon	USA				PI637833 (CMEN 698)	U.S. National Plant Germplasm System (United States Department of Agriculture, Beltsville, Maryland, USA), commercially traded as *M.* × *cordifolia*
Group I	*M.* × *cordifolia* (syn. *M.* × *villosa*) (= *M. spicata* × *M. suaveolens*)	*M.* × *cordifolia* -L.2788012				Lesser Sunda Islands	Indonesia			1968.8.5	L.2788012 (Kooy, CW 573)	Naturalis Biodiversity Center (Nationaal Herbarium Nederland (AMD, L, U, WAG), Leiden, Netherlands)
Group I	*M.* × *cordifolia* (syn. *M.* × *villosa*) (= *M. spicata* × *M. suaveolens*)	*M.* × *cordifolia* -L.2788013				Lesser Sunda Islands	Indonesia			1969.3.9	L.2788013 (Kooy, CW 586)	Naturalis Biodiversity Center (Nationaal Herbarium Nederland (AMD, L, U, WAG), Leiden, Netherlands)
Group II	*M. arvensis*	Hokushin-JP176283				Hokkaido	Japan				JP176283	Genetic Resources Center, National Agriculture and Food Research Organization (Tsukuba, Japan)
Group II	*M. arvensis*	Ryokubi-JP176285				Okayama	Japan				JP176285	Genetic Resources Center, National Agriculture and Food Research Organization (Tsukuba, Japan)
Group II	*M. arvensis*	Umahakka-JP176363					Japan				JP176363	Genetic Resources Center, National Agriculture and Food Research Organization (Tsukuba, Japan)
	M. longifolia	Horse mint-PI557757			Dijon	Côte-d'Or	France				PI557757 (CMEN 585)	Department of Horticulture, Oregon State University (Corvallis, Oregon, USA)
	M. aquatica	Numahakka-JP176358	12-1				Belgium				JP176358	Genetic Resources Center, National Agriculture and Food Research Organization (Tsukuba, Japan)
	M. × *villosa* (= *M. spicata* × *M. suaveolens*)	*M.* × *villosa* -PI558006	10071 2n US Nil.								PI558006 (CMEN 38.001)	U.S. National Plant Germplasm System (United States Department of Agriculture, Beltsville, Maryland,, USA)
Commercial cultivar												
Group I	*M. spicata* *	Spearmint [Fuj]-004										Fujita Seed Co. Ltd. (Osaka, Japan)
Group I	*M. spicata* *	Spearmint [Pot]									5900-0026	Potager Garden Co. Ltd. (Hanyu, Japan)
Group I	*Mentha* sp.	Kentucky colonel [Kur]									1426-049	Kurose Farm Kozan Lavender Hill Co. Ltd. (Sera-gun, Japan)
Group II	*Mentha* sp.	Thai mint [San]									2129-072	Sanyo Engei Co. Ltd. (Nishio, Japan)
Spearmint	*M. spicata*	Spearmint [Fuj]-009									H-93	Fujita Seed Co. Ltd. (Osaka, Japan)
Spearmint	*M. spicata*	Spearmint [Kur]									1426-517	Kurose Farm Kozan Lavender Hill Co. Ltd. (Sera-gun, Japan)
Spearmint	*M. spicata*	Spearmint [Yos]										Yoshida Engei Co. Ltd. (Konosu, Japan)
Spearmint	*M. spicata*	Spearmint-001										Local seed company (Aichi, Japan)
Spearmint	*M. spicata*	Spearmint-002										Local seed company (Aichi, Japan)
Apple mint	*M. suaveolens*	Apple mint [Mar]										Marche Aozora Co. Ltd. (Nagoya, Japan)
Apple mint	*M. suaveolens*	Apple mint [Pot]									5900-0027	Potager Garden Co. Ltd. (Hanyu, Japan)
Peppermint	*M.* × *piperita* (= *M. spicata* × *M. aquatica*)	Black peppermint									1426-052	Kurose Farm Kozan Lavender Hill Co. Ltd. (Sera-gun, Japan)
Peppermint	*M.* × *piperita* (= *M. spicata* × *M. aquatica*)	Candy mint									1426-048	Kurose Farm Kozan Lavender Hill Co. Ltd. (Sera-gun, Japan)
Peppermint	*M.* × *piperita* (= *M. spicata* × *M. aquatica*)	Curly mint										Aisai-kazoku, Agros-Kawamoto Co. Ltd. (Soraku-gun, Japan)
Peppermint	*M.* × *piperita* (= *M. spicata* × *M. aquatica*)	Eau de cologne mint									1426-045	Kurose Farm Kozan Lavender Hill Co. Ltd. (Sera-gun, Japan)

	Peppermint	*M* . × *piperita* (= *M. spicata* × *M. aquatica*)	Orange mint		Aisai-kazoku, Agros-Kawamoto Co. Ltd. (Soraku-gun, Japan)
	Peppermint	*M* . × *piperita* (= *M. spicata* × *M. aquatica*)	Peppermint	5900-0025	Potager Garden Co. Ltd. (Hanyu, Japan)
		M. longifolia	Horse mint [Sor]	HP-MNT43	Soramimi Herb Shop Co. Ltd. (Matsue, Japan)
		M. cervina	Hart’s pennyroyal mint	19810	Charm Co., Ltd. (Ora-gun, Japan)
		M. pulegium	Pennyroyal mint	1426-520	Kurose Farm Kozan Lavender Hill Co. Ltd. (Sera-gun, Japan)
		M . × *gracilis* (= *M. arvensis* × *M. spicata*)	Ginger mint		Aisai-kazoku, Agros-Kawamoto Co. Ltd. (Soraku-gun, Japan)
		M . × *smithiana* (= *M* . *aquatica* × *M. arvensis* × *M. spicata*)	Red raripila mint	HP-MNT50	Soramimi Herb Shop Co. Ltd. (Matsue, Japan)
Other Lamiaceae species					
		Nepeta sp.	Catmint	1426-101	Kurose Farm Kozan Lavender Hill Co. Ltd. (Sera-gun, Japan)

[a] Groups formed in the phylogram (Fig. 2). See Fig. 2 for the nomenclatures.

[b] Classification of each line refers to Tucker and Naczi (2006) and suppliers’ information. Unknown specific epithet and synonym are represented with "sp." and "syn.", respectively. For hybrid species, each of their predicted cross is indicated in parentheses. Red asterisk: lines, whose species classifications were suspicious, identified in this study (see Fig. 2).

[c] Mints collected in Southeast Asian countries and Japan are listed according to the countries and regions collected. The genebank accession number is indicated with a hyphen (-), and abbreviated seed company’s name is indicated in brackets for distinguishing similar names.

[d] Detailed informations are shown if known (see Supplemental Fig. 1 for their geographic locations).

[e] Accession and code nos. given to the genebank germplasms and commercial varieties, respectively, if provided.

Supplemental Table 2. Leaf morphology of 83 selected mint lines

Group[a]	Line	Leaf venation pattern[b]	Leaf shape index (LSI)[c]
Group I			
	#012	Reticulate venation type	1.5147
	#013	Reticulate venation type	1.6819
	#015	Reticulate venation type	1.5900
	#021	Reticulate venation type	1.5351
	#033	Reticulate venation type	1.5021
	#039	Reticulate venation type	1.7985
	#040	Reticulate venation type	1.4437
	#051	Reticulate venation type	1.4352
	#056	Reticulate venation type	1.5507
	#057	Reticulate venation type	1.4110
	#058	Reticulate venation type	1.3669
	#059	Reticulate venation type	1.3200
	#061	Reticulate venation type	1.6596
	#062	Reticulate venation type	1.5209
	#064	Reticulate venation type	1.8153
	#065	Reticulate venation type	1.6361
	#066	Reticulate venation type	1.4976
	#071	Reticulate venation type	1.9769
	#072	Reticulate venation type	1.8375
	#074	Reticulate venation type	1.4452
	#078	Reticulate venation type	1.6396
	#087	Reticulate venation type	1.7006
	#088	Reticulate venation type	1.5430
	#089	Reticulate venation type	1.8589
	#091	Reticulate venation type	1.6756
	#093	Reticulate venation type	1.3923
	#094	Reticulate venation type	1.5775
	#095	Reticulate venation type	1.4861
	#097	Reticulate venation type	1.5101
	#098	Reticulate venation type	1.4538
	#099	Reticulate venation type	1.4718
	#101	Reticulate venation type	1.4616
	#102	Reticulate venation type	1.6934
	#103	Reticulate venation type	1.4387
	#106	Reticulate venation type	1.6125
	#107	Reticulate venation type	1.4677
	#108	Reticulate venation type	1.2608
	#109	Reticulate venation type	1.4935
	#110	Reticulate venation type	1.4252
	#111	Reticulate venation type	1.4539
	#112	Reticulate venation type	1.5342
	#114	Reticulate venation type	1.5350
	#115	Reticulate venation type	1.4714
	#119	Reticulate venation type	1.5288
	#120	Reticulate venation type	1.5586
	#121	Reticulate venation type	1.5188
	#122	Reticulate venation type	1.6702
	#123	Reticulate venation type	1.6115

	#129	Reticulate venation type	1.6302
	#130	Reticulate venation type	1.5038
	#131	Reticulate venation type	1.5045
	#132	Reticulate venation type	1.5007
	#134	Reticulate venation type	1.5570
	#135	Reticulate venation type	1.6914
	#136	Reticulate venation type	1.5757
	#137	Reticulate venation type	1.5863
	#140	Reticulate venation type	1.7010
	#143	Reticulate venation type	1.7447
	Average (± S.D.)		1.5632 (± 0.1818) [B]
Group II			
	#007	Parallel venation type	1.8101
	#008	Parallel venation type	2.0434
	#026	Parallel venation type	1.9373
	#029	Parallel venation type	1.3871
	#034	Parallel venation type	1.8496
	#035	Parallel venation type	1.8988
	#050	Parallel venation type	1.7020
	#055	Parallel venation type	1.7870
	#073	Parallel venation type	1.8699
	#084	Parallel venation type	2.0919
	#113	Parallel venation type	2.0476
	#117	Parallel venation type	1.6645
	#118	Parallel venation type	1.9613
	#125	Parallel venation type	1.8216
	#126	Parallel venation type	1.7421
	#128A	Parallel venation type	2.0070
	#128B	Parallel venation type	2.1299
	#133	Parallel venation type	1.9458
	#138	Parallel venation type	1.9874
	Average (± S.D.)		1.8781 (± 0.2356) [A]
Spearmint			
	Spearmint-001	Reticulate venation type	1.6924
	Spearmint-002	Reticulate venation type	1.7429
	#003	Reticulate venation type	1.2100
	Average (± S.D.)		1.5485 (± 0.2632) [B]
Apple mint			
	Apple mint [KPU]	Reticulate venation type	1.4471
	Apple mint [Mar]	Reticulate venation type	1.2302
	Apple mint [Pot]	Reticulate venation type	1.2553
	Average (± S.D.)		1.3109 (± 0.1494) [C]

[a] See Fig. 2 for the nomenclature of each group.

[b] Classification of type based on visual inspection.

[c] Average value of four to five leaves in each line. Different uppercase letters indicate significant deviations ($P < 0.01$) from the other groups, as determined by the Tukey-Kramer multiple comparison test.

Supplemental Table 3. Allele numbers detected with 12 SSR markers per line and chromosome numbers of selected *Mentha* lines in this study

Group[a] \ Marker[b]	N[c]	Cont017-gene0.11	Cont028-gene0.2	Cont030-gene0.5	Cont039-gene0.9	Cont040-gene0.3	Cont055-gene0.4	Cont119-gene0.0	Cont130-gene0.5	Cont138-gene0.5	Cont171-gene0.6	EMM_007-Mp	EMM_049-Mp	Across 12 markers	Chr. no.[d]	Ref.[e]
Pennyroyal mint	1	2	2	2	1	1	1	1	1	2	2	2	1	2	20-40	
Apple mint	3	2	2	1	2	2	2	1	2	2	2	2	2	2	24 (2*x*)	Apple mint [Pot]: 2*n* = 24 (this study)
Horse mint	2	2	2	2	2	2	1	1	2	2	2	2	1	2	24 (2*x*)	Horse mint-PI557757: 2*n* = 24 (Chambers and Hummer 1994, this study)
Hart's pennyroyal mint	1	2	2	3	2	2	3	2	1	1	2	3	3	3	26, 36 (3*x*)	
M. × *villosa*	1	3	2	3	3	3	2	1	1	4	3	3	2	4	36 (3*x*), 48 (4*x*)	*M.* × *villosa*-PI558006: 2*n* = 48 (Chambers, unpublished. https://npgsweb.ars-grin.gov/gringlobal/method?id=258001)
Spearmint	6	3	3	3	3	4	3	1	2	2	2	4	3	4	48 (4*x*)	
Group I (including *M.* × *cordifolia*)	73	4	2	2	4	3	2	1	3	4	4	3	4	4	48 (4*x*)	*M.* × *cordifolia* or Kentucky colonel: 2*n* = 48 (Chambers and Hummer 1994). #056, #114, #135, Kentucky colonel [Kur], and Spearmint [Pot]: 2*n* = 48 (this study)
Group II (including cornmint)	23	7	5	6	8	6	6	2	4	6	6	5	6	8	72 (6*x*), 96 (8*x*)	Hokushin: 2*n* = 96 (Tsuda 1952). #128A and Ryokubi-JP176285: 2*n* = 96 (this study)
Water mint	1	3	2	3	4	2	2	1	2	2	3	3	2	4	96 (8*x*)	
Ginger mint	1	5	6	5	7	2	4	2	1	5	5	5	2	7	54-120	
Red raripila mint	1	7	8	4	5	3	7	2	1	2	4	2	1	8	54-120	
Peppermint	6	7	7	7	8	5	5	2	3	5	5	4	4	8	72-108	

[a] See Fig. 2 for their nomenclatures.

[b] Max allele numbers detected with each of the 12 SSR markers per line are indicated below marker names. Red letter: allele numbers corresponded to their ploidy levels.

[c] Number of lines.

[d] General chromosome numbers (2*n*) according to Chambers and Hummer (1994), Tucker and Naczi (2007), and Chambers (unpublished but are found at USDA-GRIN database, https://npgsweb.ars-grin.gov/gringlobal/method?id=258001). Ploidy levels inferred from the base chromosome number of *Mentha* (x = 12) are indicated in parentheses. For four mints (pennyroyal, water, ginger, red raripila, and pepper mints), ranges of chromosome numbers are just shown because of larger variations. Red letter: ploidy levels corresponded to their max allele numbers detected with each of the 12 SSR markers per line.

[e] Line names and chromosome numbers determined in the previous and this studies (see Supplemental Fig. 4 for photomicrographs of nine representative mint lines investigated in this study).

Supplemental Table 4. Inheritance of SSR alleles from the parents (Spearmint [Pot] and Apple mint [Pot]) to their F_1 offsprings.

Line[a] \ Marker[b]	Cont017-gene0.11				Cont028-gene0.2		Cont030-gene0.5		Cont039-gene0.9				Cont040-gene0.3				Cont055-gene0.4		Cont119-gene0.0
♀ Spearmint [Pot]	135	142	152	162	206	210	129	138	125	146	150	185		260	264	271	184	186	220
F_1 Spear × Apple-1	135	142			206	210		138	125	146		185	258	260		271	184	186	220
F_1 Spear × Apple-2		142	152		206	210		138	125	146	150			260	264	271	184	186	220
♂ Apple mint [Pot]		142			206			138	125	146			258		264			186	220

Line[a] \ Marker[b]	Cont130-gene0.5				Cont138-gene0.5				Cont171-gene0.6					EMM_007-Mp				EMM_049-Mp		
♀ Spearmint [Pot]	339		375	379	187	210	213	217	143	145	151	152			124	126	133	109	118	135
F_1 Spear × Apple-1			375		187		213	217		145		152	196	119	124		133		118	135
F_1 Spear × Apple-2	339	371	375			210	213	217		145	151		196	119			133	109	118	
♂ Apple mint [Pot]		371	375					217					196	119					118	

[a] Maternal and paternal parents (♀ and ♂, respectively) and their two F_1 offsprings (Spear × Apple-1 and 2).

[b] Allele sizes (bp) are indicated below marker names. For each combination of ♀, F_1 and ♂, alleles shared with the maternal and paternal parents are colored in red and blue, respectively. Alleles of unclear parental origins are colored in gray.

Supplemental Table 5. Genic variation statistics in five mint groups

Group[a]	N^{b}	Number of polymorphic loci within group	N_{A}^{c}	N_{E}^{d}	h^{e}	I^{f}
Group I	73*	5	1.0189 ± 0.1366	1.0012 ± 0.0094	0.0011 ± 0.0087	0.0025 ± 0.0192
Group II	23	103	1.3902 ± 0.4887	1.1443 ± 0.2513	0.0943 ± 0.1472	0.1522 ± 0.2207
Apple mint	3	20	1.0758 ± 0.2651	1.0606 ± 0.2121	0.0337 ± 0.1178	0.0482 ± 0.1687
Spearmint	6	23	1.0871 ± 0.2825	1.0562 ± 0.1976	0.0328 ± 0.1103	0.0488 ± 0.1613
Peppermint	6	92	1.3485 ± 0.4774	1.2092 ± 0.3207	0.1258 ± 0.1809	0.1892 ± 0.2661

[a] See Fig. 2 for the nomenclatures of groups I and II.

[b] Number of lines. *: including lines of suspicious classification based on the data in this study (Fig. 2, red asterisk).

[c] Observed number of alleles (mean ± standard deviation (S.D.)).

[d] Effective number of alleles (Kimura and Crow 1964) (mean ± S.D.).

[e] Nei's gene diversity (Nei 1973) (mean ± S.D.).

[f] Shannon's information index (Lewontin 1972) (mean ± S.D.).

Supplemental Table 6. Estimation of the suitable number of subpopulation (K) for structure analysis

K^a	$LnP(K)^b$		$Ln'(K)^c$	$\|Ln''(K)\|^d$	ΔK^e
	Mean	S.D.			
1	-6704.210000	0.137032	N.A.[f]	N.A.[f]	N.A.[f]
2	-4226.327273	1.219911	2477.882727	1279.925455	1049.196128
3	-3028.370000	1.612486	1197.957273	3698.827273	2293.866282
4	-5529.240000	2126.558217	-2500.870000	5251.690000	2.469573
5	-2778.420000	170.927495	2750.820000	3590.980000	21.008791
6	-3618.580000	2135.633322	-840.160000	1657.330000	0.776037
7	-2801.410000	245.338630	817.170000	1377.650000	5.615300
8	-3361.890000	1148.378710	-560.480000	2148.990000	1.871325
9	-6071.360000	2789.314147	-2709.470000	4891.320000	1.753592
10	-3889.510000	1296.443507	2181.850000	N.A.[f]	N.A.[f]

[a] Number of subpopulation.

[b] Mean and standard deviation (S.D.) of likelihood over 10 runs for each K.

[c] Mean difference between successive likelihood values of K.

[d] Absolute value of the difference between successive values of $Ln'(K)$.

[e] Mean($|L''(K)|$)/S.D.($LnP(K)$). The high value of ΔK at $K = 3$ is underlined.

[f] Not analyzed.
