# Supplementary material for: Classification of Southeast Asian mints (Mentha spp.) based on simple sequence repeat markers
Source: Breed Sci. 2022 Mar 9;72(2):181–7. doi: 10.1270/jsbbs.21058 (PMC9522532; doi:10.1270/jsbbs.21058)
Supplement: Supplementary file 3 — Supplemental Text [file 72_181_s3.pdf]

doi: 10.1270/jsbbs.21058

Supplemental Text 1.

References for interspecific hybridization and polyploidy in *Mentha* species

Ahmad, T., B.R. Tyagi, S.S. Raghuvanshi and J.R. Bahl (1992) Variation of nuclear DNA content in the genus *Mentha* L. (Lamiaceae). Cytologia 57: 359-367.

Gobert, V., S. Moja, M. Colson and P. Taberlet (2002) Hybridization in the section *Mentha* (Lamiaceae) inferred from AFLP markers. Am J Bot 89: 2017-2023.

Harley, R.M. and C.A. Brighton (1977) Chromosome numbers in the genus *Mentha* L. Bot J Linn Soc 74: 71-96.

Ikeda, N. and S. Udo (1954) Studies on mint breeding II. Karyological consideration on the phylogeny of the genus *Mentha* L. Scientific Reports of the Faculty of Agriculture, Okayama University 4: 43-49 (in Japanese with English summary).

Ikeda, N. and S. Udo (1963) Studies on the basic number of chromosomes in the genus *Mentha*. Scientific Reports of the Faculty of Agriculture, Okayama University 21: 9-15 (in Japanese with English summary).

Ikeda, N. and S. Udo (1966) Studies on *Mentha arvensis* L. Japanese Journal of Breeding 16: 251-259 (in Japanese with English summary).

Morton, J.K. (1956) The chromosome numbers of British *Menthae*. Watsonia 3: 244-252.

Ono, S. (1993) Cytogenetics of the genus *Mentha*. Scientific Reports of the Faculty of Agriculture, Okayama University 82: 69-80.

Schanzer, I.A., M.V. Semenova, O.V. Shelepova and T.V. Voronkova (2012) Genetic diversity and natural hybridization in populations of clonal plants of *Mentha aquatica* L. (Lamiaceae). Wulfenia 19: 131-139.

Tsuda, C. (1952) Fundamental studies on the breeding of mint on the somatic chromosome numbers of Japanese and Chinese peppermints. Jpn J Crop Sci 21: 178-179 (in Japanese with English summary).

Tucker, A.O. (2012) Genetics and breeding of the genus *Mentha*: A model for other

polyploid species with secondary constituents. Journal of Medically Active Plants 1: 19-29.

References for classification of *Mentha* species based on DNA markers

Apostolova, E., K. Todorov, I. Dimitrova-Dyulgerova, P. Stoyanov, R. Mladenov, G. Yahubyan and S. Naimov (2016) Analysis of the sequence of ITS1 and ITS2 regions of three *Mentha* species. Plant Genet Resour 15: 563-565.

Bunsawat, J., N.E. Elliott, K.L. Hertweck, E. Sproles and L.A. Alice (2004) Phylogenetics of *Mentha* (Lamiaceae): Evidence from chloroplast DNA sequences. Syst Bot 29: 959-964.

Capuzzo, A. and M.E. Maffei (2014) Molecular fingerprinting of some *Mentha* species by sequencing and RFLP analysis of the 5S-rRNA non-transcribed spacer region. Plant Biosyst 148: 683-690.

Capuzzo, A. and M.E. Maffei (2016) Molecular fingerprinting of peppermint (*Mentha piperita*) and some *Mentha* hybrids by sequencing and RFLP analysis of the 5S rRNA non-transcribed spacer (NTS) region. Plant Biosyst 150: 236-243.

Gobert, V., S. Moja, M. Colson and P. Taberlet (2002) Hybridization in the section *Mentha* (Lamiaceae) inferred from AFLP markers. Am J Bot 89: 2017-2023.

Jedrzejczyk, I. and M. Rewers (2018) Genome size and ISSR markers for *Mentha* L. (Lamiaceae) genetic diversity assessment and species identification. Ind Crops Prod 120: 171-179.

Khanuja, S.P.S., A.K. Shasany, A. Srivastava and S. Kumar (2000) Assessment of genetic relationships in *Mentha* species. Euphytica 111: 121-125.

Rodrigues, L., C. van den Berg, C. Póvoa and A. Monteiro (2013a) Low genetic diversity and significant structuring in the endangered *Mentha cervina* populations and its implications for conservation. Biochem Syst Ecol 50: 51-61.

Rodrigues, L., O. Póvoa, C. van den Berg, A.C. Figueiredo, M. Moldão and A. Monteiro (2013b) Genetic diversity in *Mentha cervina* based on morphological traits,

essential oils profile and ISSRs markers. Biochem Syst Ecol 51: 50-59.

Schanzer, I.A., M.V. Semenova, O.V. Shelepova and T.V. Voronkova (2012) Genetic diversity and natural hybridization in populations of clonal plants of *Mentha aquatica* L. (Lamiaceae). Wulfenia 19: 131-139.

Shasany, A.K., M.P. Darokar, S. Dhawan, A.K. Gupta, S. Gupta, A.K. Shukla, N.K. Patra and S.P.S. Khanuja (2005a) Use of RAPD and AFLP markers to identify inter- and intraspecific hybrids of *Mentha*. J Hered 96: 542-549.

Shasany, A.K., A.K. Shukla, S. Gupta, S. Rajkumar and S.P.S. Khanuja (2005b) AFLP analysis for genetic relationships among *Mentha* species. Plant Genet Resour Newsl 144: 14-19.

Theodoridis, S., A. Stefanaki, M. Tezcan, C. Aki, S. Kokkini and K.E. Vlachonasios (2012) DNA barcoding in native plants of the Labiatae (Lamiaceae) family from Chios Island (Greece) and the adjacent Çeşme-Karaburun Peninsula (Turkey). Mol Ecol Resour 12: 620-633.

Wang, H.T., X. Yu, Y. Liu, C.Y. Liang and W.L. Li (2013) Analysis of genetic variability and relationships among *Mentha* L. using the limonene synthase gene, *LS*. Gene 524: 246-252.

References cited in Supplemental Tables 3 and 5

Chambers, H.L. and K.E. Hummer (1994) Chromosome counts in the *Mentha* collection at the USDA-ARS National Clonal Germplasm Repository. Taxon 43: 423-432.

Kimura, M. and J.F. Crow (1964) The number of alleles that can be maintained in a finite population. Genetics 49: 725-738.

Lewontin, R.C. (1972). The apportionment of human diversity. *In*: Dobzhansky, T., M.K. Hecht and W.C. Steere (eds.) Evolutionary Biology. Vol. 6, Springer, New York, pp. 381-398.

Nei, M. (1973) Analysis of gene diversity in subdivided populations. Proc Natl Acad Sci USA 70: 3321-3323.
